# Lipidomics Analysis of Free Fatty Acids in Human Plasma of Healthy and Diabetic Subjects by Liquid Chromatography-High Resolution Mass Spectrometry (LC-HRMS)

**DOI:** 10.3390/biomedicines10051189

**Published:** 2022-05-20

**Authors:** Maroula G. Kokotou, Christiana Mantzourani, Charikleia S. Batsika, Olga G. Mountanea, Ioanna Eleftheriadou, Ourania Kosta, Nikolaos Tentolouris, George Kokotos

**Affiliations:** 1 Department of Chemistry, National and Kapodistrian University of Athens, 15771 Athens, Greece; mkokotou@chem.uoa.gr (M.G.K.); chrmantz@chem.uoa.gr (C.M.); cbatsika@chem.uoa.gr (C.S.B.); olgamount@chem.uoa.gr (O.G.M.); 2 Laboratory of Chemistry, Department of Food Science and Human Nutrition, Agricultural University of Athens, Iera Odos 75, 11855 Athens, Greece; 3 Center of Excellence for Drug Design and Discovery, National and Kapodistrian University of Athens, 15771 Athens, Greece; joeleftheriadou@yahoo.com (I.E.); ntentolouris@yahoo.gr (N.T.); 4 Diabetes Center, First Department of Propaedeutic and Internal Medicine, Medical School, National and Kapodistrian University of Athens, Laiko General Hospital, 15772 Athens, Greece; raniakwsta@yahoo.com

**Keywords:** diabetes, free fatty acids, hydroxy fatty acids, LC-HRMS, oxo fatty acids

## Abstract

Targeted analytical methods for the determination of free fatty acids (FFAs) in human plasma are of high interest because they may help in identifying biomarkers for diseases and in monitoring the progress of a disease. The determination of FFAs is of particular importance in the case of metabolic disorders because FFAs have been associated with diabetes. We present a liquid chromatography-high resolution mass spectrometry (LC-HRMS) method, which allows the simultaneous determination of 74 FFAs in human plasma. The method is fast (10-min run) and straightforward, avoiding any derivatization step and tedious sample preparation. A total of 35 standard saturated and unsaturated FFAs, as well as 39 oxygenated (either hydroxy or oxo) saturated FFAs, were simultaneously detected and quantified in plasma samples from 29 subjects with type 2 diabetes mellitus (T2D), 14 with type 1 diabetes mellitus (T1D), and 28 healthy subjects. Alterations in the levels of medium-chain FFAs (C6:0 to C10:0) were observed between the control group and T2D and T1D patients.

## 1. Introduction

Fatty acids (FAs) are important biomolecules that serve not only as a source of energy but also as signaling molecules and gene regulators [1,2]. Thus, they play a central role in human health and disease. FAs are found in human blood predominantly in their esterified form. However, free fatty acids (FFAs), non-esterified FAs, are also present in human blood. FFAs exert pleiotropic effects through binding to various cell-surface GPCR receptors, including free fatty acid receptors FFA1, FFA2, FFA3, and FFA4 [3]. Long-chain FFAs are ligands for FFA1 (also known as GPR40) and FFA4 (also known as GPR120), while short-chain FFAs bind to FFA2 and FFA3 receptors (also known as GPR41 and GPR43, respectively) [4,5,6]. FFA1 and FFA4 receptors may affect energy homeostasis indirectly via hormonal signaling, thus linking diet and FA intake with type 2 diabetes (T2D) prevalence [4,5,7]. FFA3 and FFA4 receptors are involved in the gut-microbiome-mediated effects on metabolic health [6].

According to various studies, altered levels of FFAs in plasma are considered both a cause and a consequence of insulin resistance and type 2 diabetes mellitus (T2D) [8,9,10]. The initial observations reported in the eighties [11], that elevated levels of FFAs in plasma are linked to T2D, have been followed by numerous studies that have explored the blood concentrations of individual FFAs in T2D subjects [12,13,14,15,16,17]. The outcome of these studies seems heterogeneous, which can be attributed to a number of reasons, including (a) the different ethnicities of the populations studied, (b) the difference in the controls selected, and (c) the presentation of the results either as absolute concentrations of each individual FFA or as the percentage of the total plasma FFAs measured. In the majority of the above studies [12,13,14,15,16], the plasma concentrations of individual FFA measured in the T2DM cohorts were all either unchanged or increased compared to the controls, while in the study of Ma et al., some FFAs were found to be decreased [17].

Adopting both targeted and untargeted liquid chromatography-high resolution mass spectrometry (LC-HRMS) analysis, we have identified the existence of new families of natural, previously unrecognized, saturated oxidized FAs, namely saturated hydroxy fatty acids (SHFAs) and saturated oxo fatty acids (SOFAs) [18,19]. We have demonstrated that hydroxystearic acids (HSAs) and hydroxypalmitic acids (HPAs), in particular 7-hydroxystearic acid, exhibit cell growth inhibitory activity and the ability to inhibit cytokine-induced β-cell apoptosis [18]. This finding raises the possibility that such FAs may have a beneficial effect, contributing to the protection of β-cell function and the prevention of autoimmune diseases, such as type 1 diabetes (T1D). Among a library of SOFAs synthesized and studied, four oxostearic acids (OSAs) were found to inhibit the cell growth of human cancer cell lines, with 6-oxostearic and 7-oxostearic acids exhibiting the highest cell growth inhibitory potency, suppressing the expression of both STAT3 and c-myc [19]. Due to the potential benefits of SHFAs and SOFAs for human health, we developed an analytical LC-HRMS method for the quantitation of these new bioactive FFAs in cow and goat milk [20,21]. Although we demonstrated the preliminary presence of free SHFAs and SOFAs in human blood [18,19], we have now carried out a detailed analysis of such oxidized FFAs in plasma. In an independent study, the chromatographic retention behavior and the detection of various SHFAs in human serum, honey and rice seedling have been reported using chemical isotope labeling-assisted LC-tandem mass spectrometry [22].

The most common method for the determination of FFAs in human plasma, employed in the majority of the studies mentioned above [12,13,14,15,16,17], was the use of gas chromatography combined with either flame ionization detection (GC-FID) or mass spectrometry detection (GC-MS), involving the conversion of FFAs into the corresponding methyl esters (FAMEs). However, an LC/MS direct method for the determination of free carboxylic acids is generally advantageous compared to the GC-MS method for the determination of their corresponding FAMEs, because it does not require any pre-treatment derivatization step. Recently, a quantitative lipidomics method for the analysis of FFAs in human plasma has been reported using a UPLC combined with an Orbitrap mass spectrometer [23]. Our group has recently developed an LC/HRMS method for the determination of 22 FFAs in cow or goat milk using a Triple-TOF mass spectrometer combined with a micro-LC, which could be easily adopted for plasma analysis [24].

It is now generally recognized that each individual FA exhibits different physiological effects, including the regulation of lipolysis and lipogenesis, endocrine signaling, and chronic inflammation [1,2,3]. Therefore, there is a great need for the quantification of each individual FA in plasma from either T2D or type 1 diabetes mellitus (T1D) subjects in comparison to control samples. The aim of the present study was the development of a straightforward LC-HRMS method for the rapid and sensitive simultaneous determination of a large set of FFAs in human plasma and the application of this method to the targeted analysis of FFAs in human samples from T2D and T1D patients and healthy controls. In addition to the usual FAs, our study aimed to detect and quantify for the first time SHFAs and SOFAs in human plasma of both healthy controls and T2D and T1D patients. In total 74 FFAs, including usual and unusual saturated and unsaturated FAs, SHFAs and SOFAs, were simultaneously detected and quantified in plasma samples from 28 healthy, 29 T2D, and 14 T1D subjects applying a rapid (10-min single run) method, avoiding any pre-treatment step.

## 2. Materials and Methods

### 2.1. Reagents and Lipid Standards

All the solvents used were of LC-MS analytical grade. Acetonitrile was purchased from Carlo Erba (Val De Reuil, France), isopropanol and methanol from Fisher Scientific (Laughborough, UK), and formic acid 98–100% from Chem-Lab (Zedelgem, Belgium). Caproic acid was purchased from Alfa Aesar (>98%, Lancashire, UK); caprylic acid, capric acid, myristic acid, myristoleic acid, pentadecanoic acid, margaric acid, linoleic acid, linolenic acid, arachidonic acid, *cis*-4,7,10,13,16,19-docosahexaenoic acid, behenic acid, and *cis*-7,10,13,16-docosatetraenoic acid from Sigma Aldrich (>99%, Steinheim, Germany); nonanoic acid, *cis*-10-heptadecenoic acid, arachidic acid, bihomo-γ-linolenic acid, *cis*-7,10,13,16,19-docosapentaenoic acid, 2-hydroxypalmitic acid (2HPA) and 2-hydroxystearic acid (2HSA) from Cayman Chemical Company (>98%, Ann Arbor, MI, USA); lauric acid from Acros Organics (>99%, Geel, Belgium); palmitic acid, 9-palmitoleic acid, stearic acid, oleic acid, and *cis*-5,8,11,14,17-eicosapentanoic acid from Fluka (analytical standard, Karlsruhe, Germany); and 16-hydroxypalmitic acid (16HPA) from Sigma-Aldrich (Darmstadt, Germany). Also, 3-Hydroxycapric acid (3HCA), 3-hydroxylauric acid (3HLA), 3-hydroxymyristic acid (3HMA), 3-hydroxypentadecanoic acid (3HPDA), 11-hydroxypalmitic acid (11HPA), 10-hydroxypalmitic acid (10HPA), 9-hydroxypalmitic acid (9HPA), 8-hydroxypalmitic acid (8HPA), 7-hydroxypalmitic acid (7HPA), 6-hydroxypalmitic acid (6HPA), 3-hydroxypalmitic acid (3HPA), 12-hydroxystearic acid (12HSA), 11-hydroxystearic acid (11HSA), 10-hydroxystearic acid (10HSA), 9-hydroxystearic acid (9HSA), 8-hydroxystearic acid (8HSA), 7-hydroxystearic acid (7HSA), 6-hydroxystearic acid (6HSA), and 3-hydroxystearic acid (3HSA) were synthesized following the general method previously described by us [18]. Finally, 14-Oxopalmitic acid (14OPA), 10-oxopalmitic acid (10OPA), 9-oxopalmitic acid (9OPA), 8-oxopalmitic acid (8OPA), 7-oxopalmitic acid (7OPA), 6-oxopalmitic acid (6OPA), 5-oxopalmitic acid (5OPA), 16-oxostearic acid (16OSA), 12-oxostearic acid (12OSA), 10-oxostearic acid (10OSA), 9-oxostearic acid (9OSA), 8-oxostearic acid (8OSA), 7-oxostearic acid (7OSA), 6-oxostearic acid (6OSA), 5-oxostearic acid (5OSA), 4-oxostearic acid (4OSA), and 3-oxostearic acid (3OSA) were synthesized at the Laboratory of Organic Chemistry, National and Kapodistrian University of Athens [19].

### 2.2. Biological Samples

Plasma samples of 28 healthy volunteers (14 males, 14 females, average age of 56.4 ± 18.9 years), 29 T2D volunteers (17 males, 12 females, average age of 67.9 ± 12.8 years), and 14 T1D volunteers (4 males, 10 females, average age of 42.9 ± 14.2 years) were collected. All subjects have provided written consent in this study, and the protocol was in accordance with the Declaration of Helsinki and Good Clinical Practice guidelines. The study was approved by the ethics committee of the Laiko General Hospital. Blood samples were collected after 8–10 h fasting. Plasma was separated at low-speed centrifugation and frozen at −20× *g* °C until analysis. None of the healthy volunteers or participants with T1D received lipid-lowering medications, while 11 (37.9%) out of 29 people with T2D received statin treatment. The demographic and clinical characteristics of the participants are shown in Supplementary Materials Table S1. 

### 2.3. Stock and Working Solutions

Stock solutions of the standard compounds (1000 mg/L in methanol) were prepared and stored at 4 °C. Working solutions (500 and 1000 ng/mL) were prepared daily by appropriate dilution.

### 2.4. Extraction of Lipids from Plasma Samples

Here, 400 μL chloroform and 200 μL methanol were added to the plasma sample (100 μL) in a screw cap glass centrifuge tube. After stirring for about 30 s, the sample was centrifuged at 4000× *g* for 10 min. The supernatant was dried under N_2_ and re-dissolved in methanol/water 1:1 in a vial and this mixture was used for the LC-MS/MS analysis.

### 2.5. LC-MS/MS Analysis

An ABSciex Triple TOF 4600 (ABSciex, Darmstadt, Germany) combined with a micro-LC Eksigent (Eksigent, Darmstadt, Germany) and an autosampler set at 5 °C and a thermostated column compartment were used to perform the LC-MS/MS measurements. Electrospray ionization (ESI) in negative mode was used for all the MS experiments. The data acquisition method consisted of a TOF-MS full scan *m/z* 50–850 and several information-dependent acquisition (IDA)-TOF-MS/MS product ion scans using 40 V collision energy (CE) with 15 V collision energy spread (CES) used for each candidate ion in each data acquisition cycle (1091). This workflow allows quantitation (primarily using TOF-MS) and confirmation (using IDA-TOF-MS/MS) in a single run. The MS resolution working conditions were: ion energy 1 (IE1) −2.3, vertical steering (VS1) −0.65, horizontal steering (HST) 1.15, and vertical steering 2 (VS2) 0.00. A Halo C18 2.7 μm, 90 Å, 0.5 × 50 mm^2^ column from Eksigent was used for the present study. The mobile phase consisted of a gradient (A: acetonitrile/0.01% formic acid/isopropanol 80/20 *v*/*v*; B: H_2_O/0.01% formic acid) and the elution gradient adopted started with 5% of phase B for 0.5 min, gradually increasing to 98% in the next 7.5 min. These conditions were kept constant for 0.5 min, and then the initial conditions (95% solvent B, 5% solvent A) were restored within 0.1 min to re-equilibrate the column for 1.5 min for the next injection (flow rate: 55 µL/min). 

### 2.6. Data Processing and Quantification

All chemical structures were drawn using ChemBioDraw Ultra 12.0 (PerkinElmer Informatics, Waltham, MA, USA). The data acquisition was carried out with MultiQuant 3.0.2 and PeakView 2.1 from (ABSciex, Darmstadt, Germany). EICs were obtained with the use of MultiQuant 3.0.2 (ABSciex, Darmstadt, Germany), which created the base peak chromatograms for the masses that achieve a 0.01 Da mass accuracy width. The relative tolerance of the retention time was set within a margin of ±2.5%. Regarding the statistical analysis, the level of significance was estimated using Excel t-Test: two-sample assuming unequal variances. 

### 2.7. Analytical Validation

#### 2.7.1. Linearity and Sensitivity

In the present study, 35 saturated and unsaturated FAs, as well as 39 SHFAs and SOFAs, were used in the present study. The full list of analytes together with their exact masses [M-H]^−^ and their chromatographic retention times R_t_ are summarized in Supplementary Materials Table S2. Limits of detection (LOD) and quantification (LOQ), including some data of our previous reports [20,21,24], are also presented in Table S2.

#### 2.7.2. Precision and Accuracy

The EU Commission decision 202/657/EC was followed as a guideline to verify the accuracy and precision of the method. Plasma samples from healthy controls were spiked at three different concentration levels to estimate the recovery and the intra-day variations. The recovery was used for the quantification of the selected compounds in plasma. The precision was investigated by means of the relative standard deviation (%RSD).

## 3. Results

### 3.1. Accuracy and Precision Data

Plasma samples were spiked with the analytes at three different concentration levels, with three replicates for each fortification level. Accuracy and precision data for SHFAs, SOFAs, and FAs are summarized in Supplementary Materials Tables S3–S5, respectively. For SHFAs, the recoveries ranged from 70 to 107 for the low spike level, from 62 to 92 for the medium spike level, and from 71 to 106 for the high spike level (Table S3). The %RSD values ranged from 0.07 to 18.07, depending on the SHFA (Table S3). For SOFAs, the recoveries ranged from 66 to 102, 62 to 85, and 67 to 86 for the low, medium, and high spike level, respectively (Table S4). The %RSD values ranged from 0.82 to 15.49 (Table S4). For FAs, the recoveries ranged from 80 to 103, 74 to 103, and 70 to 108 for the low, medium, and high spike levels, respectively (Table S5). The %RSD values ranged from 0.24 to 20.50 (Table S5).

### 3.2. Analysis of Samples

Plasma samples from 28 healthy controls and 29 T2D and 14 T1D patients were analyzed in the present study. A simple sample preparation protocol was used, involving the addition of methanol to plasma for the protein precipitation.

**Figure 1:**
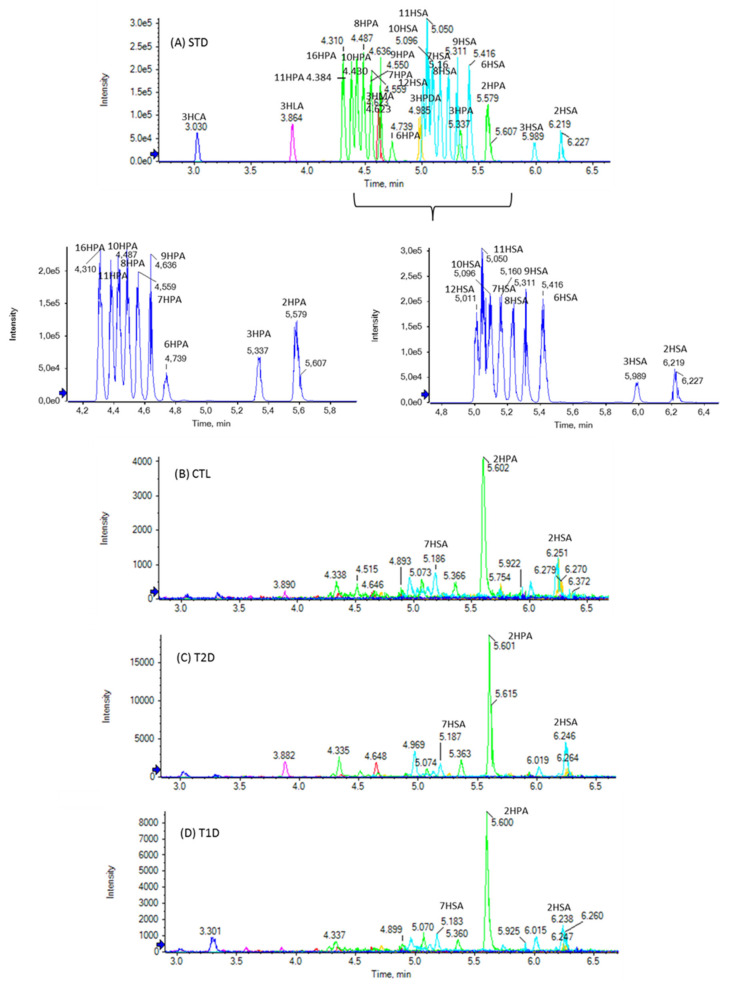
Extracted ion chromatograms (EICs) of SHFAs in a standard solution (500 ng/mL) (**A**), and in a representative control sample (**B**), a T2D sample (**C**), and a T1D sample (**D**).

**Figure 2:**
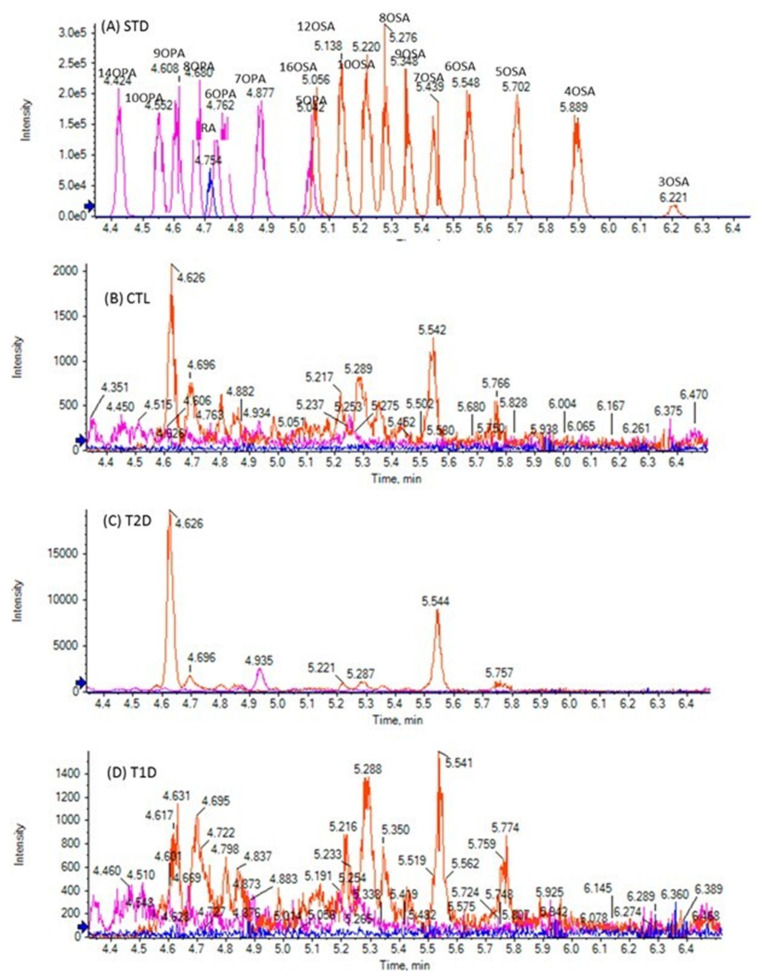
Extracted ion chromatograms (EICs) of SOFAs in a standard solution (500 ng/mL) (**A**), and in a representative control sample (**B**), T2D sample (**C**) and T1D sample (**D**).

**Figure 3:**
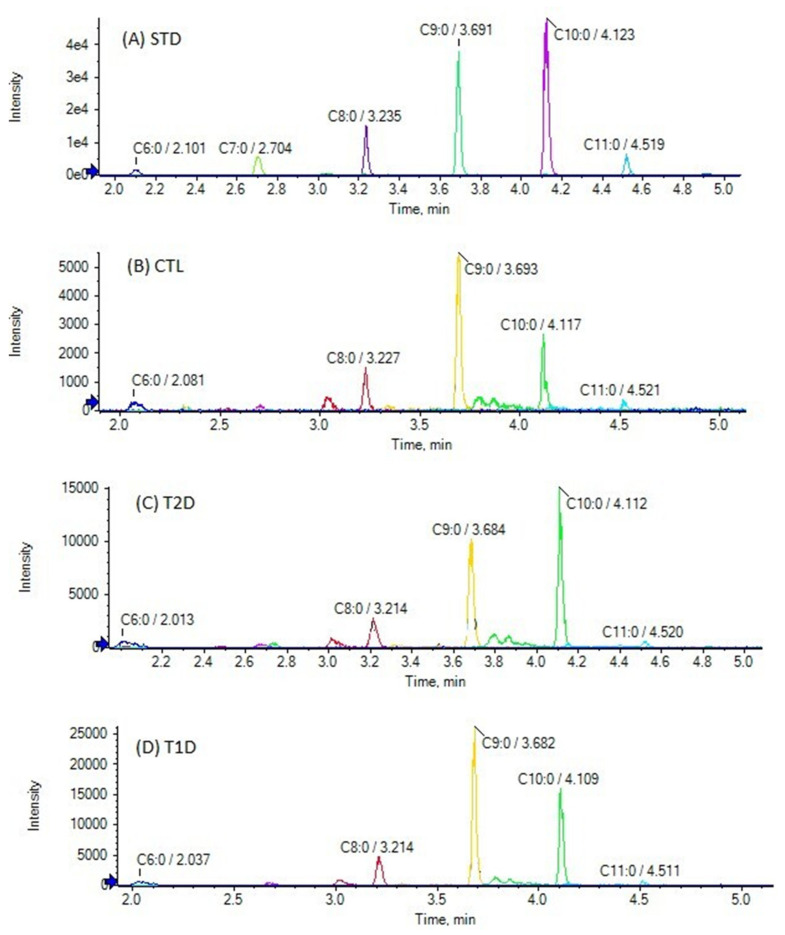
Extracted ion chromatograms (EICs) of medium-chain FAs (C6:0- C11:0) in a standard solution (500 ng/mL) (**A**), and in a representative control sample (**B**), a T2D sample (**C**), and a T1D sample (**D**).

**Figure 4:**
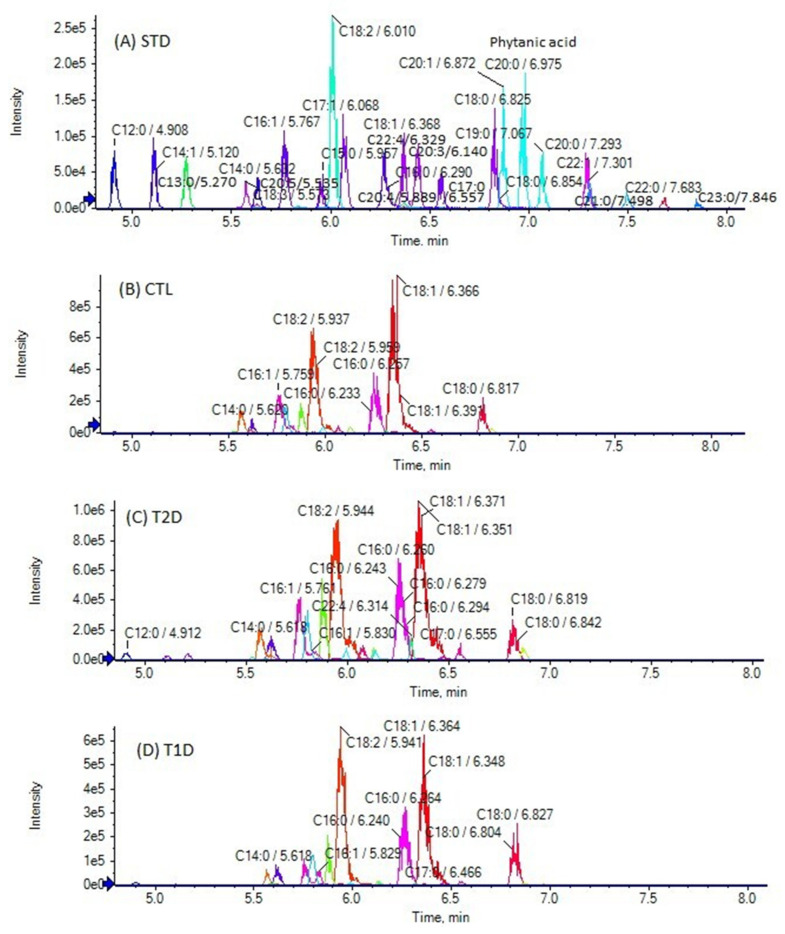
Extracted ion chromatograms (EICs) of FAs in a standard solution (500 ng/mL) (**A**), and in a representative control sample (**B**), a T2D sample (**C**) and a T1D sample (**D**).

The chromatographic method used allows the simultaneous determination of FFAs in a 10-min single run. The extracted ion chromatogram (EIC) of SHFAs in a standard solution (A), as well as EICs of a representative control (B), a T2D (C), and a T1D (D) sample are presented in Figure 1. As shown in Figure 1A, the reference isobaric SHFAs were distinctly separable with this chromatographic technique. Similarly, Figure 2 shows the EIC of SOFAs in a standard solution (A), as well as EICs of representative control (B), T2D (C), and T1D (D) samples. Figure 3 and Figure 4 also present EICs of standard solutions of FAs and representative control, T2D, and T1D samples. Supplementary Materials Figure S1 illustrates the multi-sample analysis data for medium-chain FAs in T2D samples (A) and TID samples (B), while in Figure S2 an extracted ion chromatogram (EIC) of FAs in a standard solution (500 ng/mL) is presented.

The contents of the 74 analytes in plasma samples (in triplicate) are summarized in Table 1, Table 2 and Table 3. The contents of FFAs are expressed as nmol per mL of plasma. The data expressed as the percentage of total FFAs analyzed are shown in Supplementary Materials Table S6.

Ten different SHFAs were detected and quantified in healthy controls (Table 1). 2HPA was estimated as the most abundant (0.19 ± 0.04 nmol/mL), followed by 2HSA and 3HSA (0.09 ± 0.03 nmol/mL and 0.04 ± 0.01 nmol/mL, respectively). 3HCA, 3HLA, 16HPA, 11HPA, 3HPA, 7HSA, and 8HSA were estimated at lower concentrations (0.02 to 0.01 nmol/mL), while although 3HMA, 3HPDA, 10HPA, 9HPA, 8HPA, 7HPA, 6HPA, 12HSA, 11HSA, 10HSA, 9HSA, and 6HSA were detected, their concentrations were lower than their LOQs. The concentration of SHFAs in T2D and T1D samples were similar to those of healthy controls, with the exception of 7HSA, which was found elevated in some samples (mean value 0.11 ± 0.50 nmol/mL).

One SOFA was quantified in the samples of healthy controls, T2D and T1D patients, namely 6OSA, at concentrations of 0.04 ± 0.02 nmol/mL, 0.06 ± 0.03 nmol/mL, and 0.05 ± 0.03 nmol/mL, respectively (Table 2). Three additional SOFAs, 10OSA, 9OSA, and 4OSA, were quantified in T2D patients at 0.01 nmol/mL concentration, while 10OSA and 9OSA were also found in T1D patients at 0.01 nmol/mL concentration. For all the rest of the SOFAs studied, although they were detected, their concentrations were lower than their LOQs. The isobaric to OSA hydroxy monounsaturated ricinoleic acid was found in healthy controls, T2D and T1D patients at similar concentrations, ranging from 0.02 to 0.04 nmol/mL (Table 3).

In healthy controls, the long-chain saturated FAs C14:0, C16:0, and C18:0, as well as the unsaturated FAs C16:1, C18:1, and C18:2 were found to be the most abundant FFAs, at concentrations higher than 10 nmol/mL (Table 3). The contents of C14:0, C16:0, C18:0, C16:1, C18:1 and C18:2 were estimated at mean values of 12.63 ± 7.76 nmol/mL, 66.44 ± 15.23 nmol/mL, 18.92 ± 4.94 nmol/mL, 10.08 ± 7.49 nmol/mL, 38.91 ± 19.56 and 136.29 ± 84.23, respectively. 

The long-chain saturated odd-chain FA C17:0, as well as the unsaturated C18:3, C20:4, C22:4 and C22:5 were estimated at mean values of 2.94 ± 2.66 nmol/mL, 1.16 ± 0.45 nmol/mL, 1.18 ± 0.31 nmol/mL, 2.15 ± 1.89 nmol/mL, and 1.80 ± 1.64 nmol/mL, respectively (Table 3). Several other long-chain FAs were quantified at concentrations lower than 1 nmol/mL: C12:0 (0.68 ± 0.99), C13:0 (0.04 ± 0.01), C14:1 (0.59 ± 0.70), C15:0 (0.30 ± 0.13), C17:1 (0.79 ± 0.81), C19:0 (0.03 ± 0.02), C20:0 (0.21 ± 0.07), C20:1 (0.80 ± 0.72), phytanic (0.03 ± 0.02), C20:5 (0.67 ± 0.66), C22:1 (0.04 ± 0.03), and C24:1 (0.40 ± 0.29). C20:3, C21:0, C23:0, C22:6 and C22:0 were detected, but not quantified, because their contents were lower than their LOQs.

**Figure 5:**
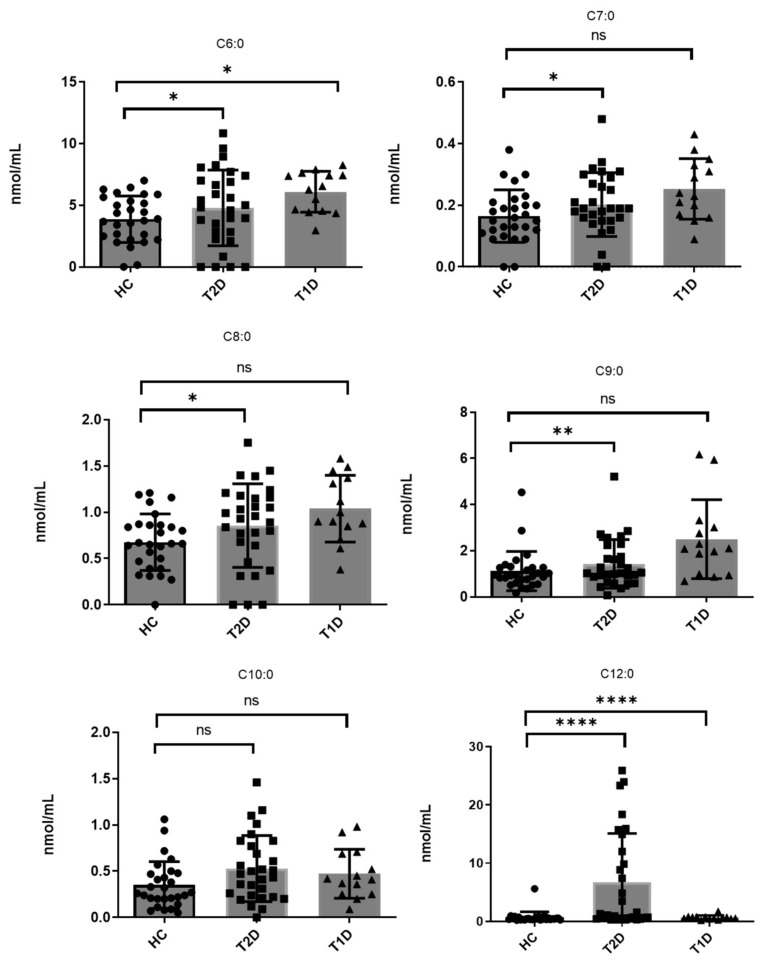
Comparison of plasma concentrations (nmol/mL) of C6:0, C7:0, C8:0, C9:0, C10:0 and C12:0 between healthy controls and T2D and T1D patients. Graphs were created using GraphPad Prism 9.2.0. One-way ANOVA statistical analysis was performed for each separate set comparing to control. ns (not significant): *p* > 0.05. * *p* < 0.05, ** *p* < 0.01, **** *p* < 0.0001.

As summarized in Table 3, all these long-chain FAs were also quantified in the samples from T2D and T1D patients, and their contents were found in general at approximately similar levels. C12:0 attracts some attention because it appears elevated in T2D patients, as shown in Figure 5.

The contents of medium-chain saturated FAs (C6:0 to C11:0) in the plasma of T2D and T1D patients, in comparison to healthy controls, stood out as notable. In both healthy controls and diabetic subjects, C6:0 and C9:0 were found to be the most abundant. In controls, C6:0 and C9:0 were found at mean values of 3.87 ± 1.89 nmol/mL and 1.12 ± 0.85 nmol/mL, respectively (Table 3). C8:0, C10:0 and C7:0 were found at lower concentrations (0.68 ± 0.30 nmol/mL, 0.35 ± 0.25 nmol/mL, 0.16 ± 0.08 nmol/mL, respectively), while C11:0 was detected at levels lower than its LOQ. Interestingly, the levels of these medium-chain FAs were found elevated in both T2D and T1D patients. In T2D patients, C6:0, C9:0, C8:0, C10:0, and C7:0 were estimated at mean values of 4.79 ± 3.07, 1.43 ± 0.03, 0.86 ± 0.45, 0.53 ± 0.36, and 0.20 ± 0.10 nmol/mL, respectively. In addition, C11:0 was found at a low concentration of 0.02 ± 0.03 nmol/mL. In T1D patients, C6:0, C9:0, C8:0, C10:0, and C7:0 were estimated at mean values of 6.09 ± 1.67, 2.50 ± 1.71, 1.04 ± 0.36, 0.47 ± 0.26, and 0.25 ± 0.09 nmol/mL, respectively. The alterations of the levels of these medium-chain FFAs in healthy controls, T2D, and T1D patients are better demonstrated in Figure 5. In addition, the levels of the rest FFAs are shown in Supplementary Materials Figure S3.

## 4. Discussion

The contents of FFAs in human plasma may be expressed either as a percentage of total FFAs or as a concentration of each particular FFA. As shown by Chilton et al. [25], the correlation of disease biomarkers (such as blood lipids) or disease risk with levels of circulating or cellular FAs may lead to dissimilar correlations, depending on the expression of FFAs levels, thus impacting the interpretation of the results. In the present work, we estimate the concentration of each particular FFA as nmol/mL.

A total of 39 oxidized (hydroxy and oxo) saturated FFAs were studied in the plasma of healthy controls, T2D, and T1D patients. In healthy controls, among the 22 different free SHFAs studied, 2HPA, 2HSA, and 3HSA were found at concentrations varying from 0.19 ± 0.04 nmol/mL to 0.04 ± 0.01 nmol/mL. In addition, 3HCA, 3HLA, 16HPA, 11HPA, 3HPA, 7HSA, and 8HSA were determined at lower concentrations, ranging from 0.02 to 0.01 nmol/mL. A number of 3-hydroxy FFAs have been previously analyzed in plasma of healthy controls and patients with known defects of mitochondrial fatty acid β-oxidation, using a GC-MS method after trifluoroacetylation of hydroxyl groups and *tert*-butyldimethylsilylation of the carboxyl groups [26]. The levels of literature values are shown in Table 1 for comparison. 2-Hydroxy FAs have been also previously determined as *tert*-butyldimethylsilyl derivatives in plasma by GC-MS and 2HSA has been found at a 0.14 ± < 0.01 concentration [29]. For the first time in this work, some HSAs and HPAs carrying the hydroxyl group at positions higher than 2 and 3, namely 7HSA, 8HSA, 11HPA, and 16HPA, were identified and quantified in plasma. We have recently demonstrated that HSAs and HPAs, in particular 7HSA, exhibit cell growth inhibitory activity and the ability to inhibit the cytokine-induced β-cell apoptosis [18]. Thus, their presence in human plasma may be of biological relevance and worthy of further investigation. Additionally, HSAs and HPAs are precursors of fatty acid esters of hydroxy fatty acids (FAHFAs), a recently discovered class of bioactive lipids exhibiting antidiabetic and anti-inflammatory activity [30]. Up to now, the detailed pathways for the biosynthesis of FAHFAs are not known, however, it has been shown that the gavage of mice with 9-hydroxyheptadecanoic acid resulted in the synthesis of FAHFAs containing this fatty acid, indicating that HSAs may be converted in vivo in FAHSAs (28). We have shown that HSAs and HPAs are natural FAs found in milk [20]. Thus, the presence of such SHFAs (7HSA, 8HSA, 11HPA, and 16HPA) in human blood may be explained either by food ingestion or by oxidation of saturated FAs by an unknown up to now mechanism. Although an enzyme able to introduce a hydroxyl group at position 2, namely fatty acid 2-hydroxylase, is known [31], no enzymatic pathway for hydroxylation at other positions of a long chain has been revealed. The levels of SHFAs in T2D and T1D patients are generally similar. Only 7HSA was found somewhat elevated in T2D patients (0.11 versus 0.01 nmol/mL). However, such alterations were not clear because, in several healthy controls and T2D patients, the levels of SHFAs were lower than their corresponding LOQs.

Several OSAs and OPAs were detected in the plasma of healthy controls. However, only 6OSA was quantified at a mean value of 0.04 ± 0.02 nmol/mL. This particular oxidized FA was also quantified in T2D and T1D patients but significant alterations were not observed. In addition, 10OSA and 9OSA were quantified in both T2D and T1D patients, while 4OSA was quantified only in T2D patients. However, again these alterations were not clear because in several healthy controls and T2D and T1D patients, the levels of SOFAs were lower than their corresponding LOQs. To our knowledge, ricinoleic acid (isobaric to OSA) was detected and quantified for the first time in the plasma of healthy controls, T2D, and T1D patients, at similar levels. Its presence in human blood may be explained by the consumption of milk and other dairy products. As we previously demonstrated [21], ricinoleic acid was found in milk, and its presence may be attributed to animal feeding containing castor oil supplements.

As presented in Table 3, the levels of the most abundant saturated and unsaturated FFAs (C14:0, C16:0, C18:0, C16:1, C18:1, and C18:2) in healthy controls were found at concentrations more or less similar to those reported in the literature [12,13,28]. Dennis et al. have previously analyzed FFAs in plasma obtained from healthy individuals after overnight fasting and with a gender balance and an ethnic distribution that is representative of the US population, by GC/MS after derivatization to pentafluorobenzyl esters [28], while other studies have compared the concentrations of healthy controls and diabetic patients [12,13]. All these literature values for FFAs are included in Table 3 for comparison. The differences observed may be attributed to various factors, including ethnicities and dietary habits. 

In our study, for the majority of usual long-chain FFAs, no significant alterations were observed between either T2D or T1D patients and healthy controls. However, special attention has to be paid to medium-chain FFAs, consisting of 6 to 10 carbon atoms. As shown in Figure 5, the most significant alterations were observed for C6:0 in both T2D and T1D patients and for C7:0, C8:0, and C9:0 in T2D patients. In all cases, increased levels were observed in T2D and T1D patients.

Limited data on the biological importance of medium-chain FFAs are available in the literature and a systematic study on their levels and potential role in diabetes is missing. C6:0 has been recently determined in human serum of healthy controls by LC-MS/MS, after derivatization with 2-nitrophenylhydrazine, and its concentration was found to be 2.0 ± 2.5 nmol/mL [27], which can be considered comparable to the estimated mean value 3.87 ± 1.89 nmol/mL of the present study. In our study, a considerable increase was found in diabetic patients, in particular in T1D patients (mean value 6.09 ± 1.67 nmol/mL). Medium-chain FAs, including C6:0, have been reported to promote TH1 and TH17 differentiation, thus supporting inflammation [32]. Most recently, C6:0 has been associated with multiple sclerosis, playing a role in chronic inflammation [33]. Thus, the elevation of C6:0 in T2D and T1D patients may reflect an underlying chronic inflammatory condition. In a recent study on the long-term future onset of CAD in Japanese diabetic patients, C9:0 has been identified in human serum samples, using capillary electrophoresis (CE-TOFMS) method of non-targeted analysis, as one out of seven metabolites, the levels of which were significantly altered [34]. However, no data on alteration of its levels have been reported. A multiplatform metabolomics study in an epidemiological setting has revealed a series of deregulated metabolites that associate with diabetes [35]. C6:0, C7:0, and C9:0 are included in the deregulated metabolites. A non-targeted metabolomics approach looking for biomarkers for T2D and impaired fasting glucose has also indicated some medium-chain FAs as potential biomarkers [36]. However, none of these studies provide data on the concentrations of medium-chain FAs in healthy controls and diabetic patients. 

The physiological contributions of each one of the diverse FFAs and how their levels change depending on the metabolic or the cardiovascular disorder remain largely unknown. Accumulating experimental data enforce the notion that the pharmacological effects of dietary FAs in metabolic and cardiovascular disorders have to be classified in a better way based on their biological rather than their chemical function [37]. The application of a straightforward in-depth lipidomics analysis of a big set of FFAs in human plasma is a first step to identifying and studying alterations in T2D and T1D patients. The proposed LC-HRMS method also provides the possibility of untargeted or suspect screening of additional FFA species, due to the high accuracy offered by the HRMS detector. The present study suggests that medium-chain FFAs are potential biomarkers of metabolic diseases. However, further studies involving big control and diabetic patient groups will be necessary to evaluate the importance of medium-chain FFAs as potential biomarkers of diabetes and as risk prediction for future consequences.

## 5. Conclusions

In conclusion, we present an LC-HRMS method, which permits the detection and quantification of a large set of FFAs in human plasma, in a short time, avoiding any tedious sample pre-treatment. For the first time, the presence of 39 different oxidized saturated FAs, hydroxy and oxo FAs, was studied in detail in human plasma. In addition to 2- and 3-HSA and HPA; 7HSA, 8HSA, 11HPA and 16HPA, and one oxo fatty acid, 6OSA were quantified in human plasma. Moreover, 35 usual FAs were also detected and quantified in human plasma of healthy controls and T2D and T1D patients. Interesting alterations in the levels of medium-chain FAs (C6:0 to C10:0) were observed between the healthy controls and participants with diabetes mellitus, suggesting that these FAs deserve further investigation. Thus, the proposed targeted method may find further applications for studying human plasma samples from patients suffering from other diseases, broadening our knowledge of the human lipidome and the alterations of various lipids associated with the development and progression of not only diabetes but also other diseases.

## Figures and Tables

**Table 1:** Contents of free SHFAs in plasma samples (nmol/mL).

Analyte	Control Plasma Sample (Number of Samples 28)				Literature (nmol/mL)	T2D Plasma Sample (Number of Samples 29)					T1D Plasma Sample (Number of Samples 14)			
	Minimum Value (nmol/mL)	Maximum Value (nmol/mL)	Mean Value ± SD (nmol/mL)	*α*		Minimum Value (nmol/mL)	Maximum Value (nmol/mL)		Mean Value ± SD (nmol/mL)	*α*	Minimum Value (nmol/mL)	Maximum Value (nmol/mL)	Mean Value ± SD (nmol/mL)	*α*
3HCA	<LOQ *^a^*	0.07	0.02 ± 0.01 *^b^*	***	0.15 [25]	<LOQ *^c^*	0.29		0.03 ± 0.02 *^b^*	**	<LOQ *^d^*	0.04	0.01 ± 0.01 *^b^*	**
3HLA	<LOQ *^a^*	0.05	0.01 ± 0.01 *^b^*	**	0.07 [25]	<LOQ *^a^*	0.10		0.02 ± 0.02 *^b^*	***	<LOQ *^e^*	0.03	0.01 ± 0.01 *^b^*	NS
3HMA, 3HPDA	<LOQ			-		<LOQ				-	<LOQ			-
16HPA	<LOQ *^f^*	0.05	0.01 ± 0.01 *^b^*	**						-				-
11HPA	<LOQ *^c^*	0.04	0.01 ± 0.01 *^b^*	*						-				-
10HPA, 9HPA, 8HPA, 7HPA, 6HPA	<LOQ			-						-				-
3HPA	<LOQ *^g^*	0.04	0.02 ± 0.01 *^b^*	***	0.18 [25]	0.02		0.09	0.03 ± 0.01	***	0.02	0.04	0.02 ± 0.01	***
2HPA	<LOQ *^d^*	0.38	0.19 ± 0.04 *^b^*	***	0.14 [26]	0.10		0.49	0.25 ± 0.06	***	0.11	0.31	0.21 ± 0.01	***
12HSA, 11HSA, 10HSA, 9HSA, 6HSA	<LOQ			-		<LOQ				-	<LOQ			-
7HSA	<LOQ *^f^*	0.03	0.01 ± 0.01 *^b^*	**		<LOQ *^a^*		2.78	0.11 ± 0.50 *^b^*	NS	<LOQ *^g^*	0.06	0.02 ± 0.01 *^b^*	**
8HSA	<LOQ *^c^*	0.03	0.01 ± 0.01 *^b^*	NS		<LOQ				-	<LOQ			-
3HSA	0.02	0.08	0.04 ± 0.01	***	0.02 [25]	0.03		0.14	0.05 ± 0.02	***	0.03	0.06	0.04 ± 0.01	***
2HSA	0.03	0.24	0.09 ± 0.03	***	0.14 [26]	0.04		0.34	0.11 ± 0.03	***	0.02	0.12	0.09 ± 0.03	***

Content lower than LOQ in *^a^* 16, *^c^* 22, *^d^* 10, *^e^* 12, *^f^* 20, *^g^* 7 samples; *^b^* the mean value was determined using medium-bound approach; <LOQ: lower of limit of quantification; SD: standard deviation; *α*: level of significance; NS: *p* > 0.05. * *p* < 0.05, ** *p* < 0.01,*** *p* < 0.001.

**Table 2:** Contents of free SOFAs in plasma samples (nmol/mL).

Analyte	Control Plasma Sample (Number of Samples 28)				T2D Plasma Sample (Number of Samples 29)				T1D Plasma Sample (Number of Samples 14)			
	Minimum Value (nmol/mL)	Maximum Value (nmol/mL)	Mean Value ± SD (nmol/mL)	*α*	Minimum Value (nmol/mL)	Maximum Value (nmol/mL)	Mean Value ± SD (nmol/mL)	*α*	Minimum Value (nmol/mL)	Maximum Value (nmol/mL)	Mean Value ± SD (nmol/mL)	*α*
14OPA, 10OPA, 9OPA, 8OPA, 7OPA, 6OPA, 5OPA, 16OSA, 12OSA, 8OSA, 7OSA, 5OSA, 3OSA	<LOQ			-	<LOQ			-	<LOQ			-
10OSA				-	<LOQ *^a^*	0.02	0.01 ± 0.02 *^b^*	NS	<LOQ *^c^*	0.02	0.01 ± 0.02 *^b^*	NS
9OSA				-	<LOQ *^a^*	0.02	0.01 ± 0.01 *^b^*	NS	<LOQ *^c^*	0.03	0.01 ± 0.01 *^b^*	*
6OSA	<LOQ *^d^*	0.10	0.04 ± 0.02 *^b^*	***	0.03	0.22	0.06 ± 0.03	***	0.02	0.11	0.05 ± 0.03	***
4OSA	<LOQ			-	<LOQ *^a^*	0.02	0.01 ± 0.01 *^b^*	**	<LOQ			-

Content lower than LOQ in *^a^* 24, *^c^* 10, *^d^* 5, *^b^* the mean value was determined using medium-bound approach; <LOQ: lower of limit of quantification; SD: standard deviation; *α*: level of significance; NS: *p* > 0.05. * *p* < 0.05, ** *p* < 0.01,*** *p* < 0.001.

**Table 3:** Contents of FFAs in plasma samples (nmol/mL).

Analyte	Control Plasma Sample (Number of Samples 28)				Literature (nmol/mL)	T2D Plasma Sample (Number of Samples 29)				T1D Plasma Sample (Number of Samples 14)			
	Minimum Value (nmol/mL)	Maximum Value (nmol/mL)	Mean Value ± SD (nmol/mL)	*α*		Minimum Value (nmol/mL)	Maximum Value (nmol/mL)	Mean Value ± SD (nmol/mL)	*α*	Minimum Value (nmol/mL)	Maximum Value (nmol/mL)	Mean Value ± SD (nmol/mL)	*α*
C6:0	0.16	6.44	3.87 ± 1.89	***	2.0 ± 2.5 [27]	<LOQ *^a^*	9.62	4.79 ± 3.07 *^b^*	***	2.97	8.25	6.09 ± 1.67	***
C7:0	<LOQ *^c^*	0.38	0.16 ± 0.08 *^b^*	***		<LOQ *^c^*	0.48	0.20 ± 0.10 *^b^*	***	0.09	0.43	0.25 ± 0.09	***
C8:0	<LOQ *^c^*	1.21	0.68 ± 0.30 *^b^*	***		<LOQ *^c^*	1.45	0.86 ± 0.45 *^b^*	***	0.61	1.58	1.04 ± 0.36	***
C9:0	0.16	4.53	1.12 ± 0.85	***		0.07	5.21	1.43 ± 0.03	***	0.99	5.94	2.50 ± 1.71	***
C10:0	0.05	1.06	0.35 ± 0.25	***		<LOQ *^c^*	1.46	0.53 ± 0.36 *^b^*	***	0.20	0.98	0.47 ± 0.26	***
C11:0	<LOQ			-		<LOQ *^d^*	0.09	0.02 ± 0.03 *^b^*	***	<LOQ			-
C12:0	0.25	5.61	0.68 ± 0.99	***	0.719 ± 0.029 [28] 1.10 ± 0.55 [13]	0.30	25.92	6.75 ± 8.35	***	0.25	1.73	0.67 ± 0.36	***
C13:0	0.02	0.07	0.04 ± 0.01	***		0.02	0.16	0.06 ± 0.03	***	0.04	0.10	0.06 ± 0.02	***
C14:0	0.74	29.97	12.63 ± 7.76	***	13.2 ± 6.8 [12] 4.56 ± 2.06 [13] 2.93 [15] 6.06 ± 0.069 [28]	1.97	32.58	13.72 ± 7.38	***	4.76	22.60	15.19 ± 7.11	***
C14:1	<LOQ *^c^*	2.55	0.59 ± 0.70 *^b^*	***		<LOQ *^c^*	3.12	0.57 ± 0.73 *^b^*	***	0.07	2.99	0.75 ± 0.89	***
C15:0	0.12	0.40	0.30 ± 0.13	***	0.653 ± 0.004 [28] 0.93 ± 0.26 [13]	0.12	1.44	0.42 ± 0.24	***	0.07	2.99	0.75 ± 0.89	***
C16:0	16.34	108.20	66.44 ± 15.23	***	163.9 ± 53.2 [12] 135.5 ± 25.14 [13] 92.7 [15] 63.8 ± 0.4 [28]	29.61	121.48	72.80 ± 14.93	***	52.72	111.92	78.74 ± 17.25	***
C16:1	0.12	22.26	10.08 ± 7.49	***	26.1 ± 11.3 [12] 7.70 ± 4.17 [13] 14.7 ± 0.169 [28]	0.45	24.16	8.40 ± 6.78	***	2.88	22.47	10.83 ± 6.40	***
C17:0	0.33	10.32	2.94 ± 2.66	***	3.2 ± 1.2 [12] 1.20 ± 0.003 [28]	0.15	11.58	3.29 ± 2.98	***	0.68	6.09	2.86 ± 1.62	***
C17:1	0.01	2.28	0.79 ± 0.81	***	1.03 ± 0.057 [28]	0.01	3.79	0.85 ± 0.98	***	0.10	2.41	0.86 ± 0.74	***
C18:0	5.38	26.09	18.92 ± 4.94	***	50.7 ± 21.1 [12] 46.33 ± 9.82 [13] 38.7 [12] 22.1 ± 0.035 [28]	8.40	34.11	16.29 ± 8.48	***	10.35	35.89	22.68 ± 7.60	***
C18:1	6.49	69.45	38.91 ± 19.56	***	160.6 ± 46.7 [12] 117.20 ± 31.77 [13] 45.1 [12] 80.3 ± 9.33 [28]	8.11	79.98	40.94 ± 20.04	***	22.00	67.21	46.15 ± 12.36	***
C18:2	3.28	288.79	136.29 ± 84.23	***	73.4 ± 18.4 [12] 131.68 ± 32.03 [13] 54.8 [15] 15.2 ± 0.437 [28]	1.44	324.44	109.50 ± 85.10	***	29.99	333.60	142.44 ± 79.52	***
C18:3	0.01	2.90	1.16 ± 0.45	***	3.9 ± 1.6 [12] 5.01 ± 1.82 [13] 1.11 ± 0.005 [28]	0.04	3.02	1.35 ± 0.71	***	0.16	4.23	1.49 ± 0.65	***
Ricinoleic acid	<LOQ *^e^*	0.15	0.03 ± 0.02 *^b^*	**	<LOQ *^e^*	0.27	0.04 ± 0.02 *^b^*	**	<LOQ*^d^*	0.08	0.02 ± 0.03 *^b^*	**	RA
C19:0	0.01	0.09	0.03 ± 0.02	***	0.05 [15]	<LOQ *^e^*	0.10	0.02 ± 0.02 *^b^*	***	0.01	0.07	0.04 ± 0.01	***
C20:0	0.10	0.38	0.21 ± 0.07	***	0.238 ± 0.002 [28] 1.73 ± 0.78 [13] 0.12 [15]	0.06	0.37	0.22 ± 0.09	***	0.10	0.25	0.19 ± 0.06	***
C20:1	0.01	2.84	0.80 ± 0.72	***		0.01	4.05	0.92 ± 0.90	***	0.12	1.63	0.70 ± 0.62	***
C20:3	<LOQ			-	0.542 ± 0.005 [28]	<LOQ			-	<LOQ			-
C20:4	0.02	2.77	1.18 ± 0.31	***	5.7 ± 3.5 [12] 23.14 ± 7.90 [13] 2.94 ± 0.058 [28]	0.20	2.79	1.21 ± 0.40	***	0.60	2.29	1.11 ± 0.35	***
C20:5	0.02	6.90	0.67 ± 0.66	***	0.4 ± 0.4 [12] 1.57 ± 0.75 [13] 0.435 ± 0.010 [28]	0.11	7.31	1.01 ± 1.36	***	0.21	2.09	0.74 ± 0.46	***
Phytanic acid	<LOQ *^c^*	0.08	0.03 ± 0.02 *^b^*	***		<LOQ *^c^*	0.17	0.05 ±0.05 *^b^*	***	0.01	0.10	0.05 ± 0.03	***
C21:0	<LOQ			-		<LOQ			-	<LOQ			-
C22:0	<LOQ			-	0.160 ± 0.007 [28]	<LOQ			-	<LOQ			-
C22:1	0.01	0.11	0.04 ± 0.03	***	0.028 ± 0.002 [28]	0.02	0.18	0.05 ± 0.03	***	0.01	0.10	0.04 ± 0.02	***
C22:4	0.02	6.90	2.15 ± 1.89	***	1.34 ± 0.64 [13] 0.364 ± 0.005 [28]	0.01	8.83	2.17 ± 2.38	***	0.40	3.39	1.41 ± 0.89	***
C22:5	<LOQ *^a^*	0.10	1.80 ± 1.64 *^b^*	***	0.1 ± 0.2 [12] 1.82 ± 1.24 [13] 0.400 ± 0.005 [28]	<LOQ *^d^*	6.01	0.91 ± 1.46 *^b^*	***	<LOQ *^a^*	2.35	0.94 ± 0.87 *^b^*	***
C22:6	<LOQ			-	0.990 ± 0.009 [28] 0.6 ± 0.7 [12] 7.81 ± 2.94 [13]	<LOQ				<LOQ			
C23:0	<LOQ			-	0.033 ± 0.004 [28]	<LOQ			-	<LOQ			-
C24:1	0.02	1.11	0.40 ± 0.29	***		0.01	2.45	0.38 ± 0.42	***	0.08	0.67	0.31 ± 0.19	***

Content lower than LOQ in *^a^* 4, *^c^* 2, *^d^* 18, *^e^* 12 samples; *^b^* the mean value was determined using medium-bound approach; <LOQ: lower of limit of quantification; SD: standard deviation; *α*: level of significance; NS: *p* > 0.05. ** *p* < 0.01,*** *p* < 0.001.

## Data Availability

All data supporting this study are included in the article and Supplementary Materials.

## Supplementary Materials

The following supporting information can be downloaded at: https://www.mdpi.com/article/10.3390/biomedicines10051189/s1, Table S1: Demographic and clinical characteristics of the participants; Table S2: List of analytes together with their exact masses [M-H]^−^, their chromatographic retention times R_t_, and their limits of detection (LOD) and quantification (LOQ); Table S3: Accuracy (recovery %) and precision data (RSD %) in spiked plasma samples for SHFAs; Table S4: Accuracy (recovery %) and precision data (RSD %) in spiked plasma samples for SOFAs; Table S5: Accuracy (recovery %) and precision data (RSD %) in spiked plasma samples for FAs; Table S6: Percentage of total FFAs analyzed; Figure S1: Multi sample analysis of medium-chain fatty acids (C6:0–C12:0) (A) T1D and (B) T2D; Figure S2: Extracted ion chromatograms (EICs) of FAs in a standard solution (500 ng/mL); Figure S3: Comparison of plasma concentrations (nmol/mL) of 3HSA, 2HSA, C13:0, C14:0, C15:0, C16:0, C16:1, C17:0, C17:1, C18:0, C18:1, C18:2, C18:3, C20:0, C20:1, C20:4, C20:5, C22:1, C22:4, and C24:1 between healthy controls and T2D and T1D patients. Graphs were created using GraphPad Prism 9.2.0. References [20,21,24] are cited in the supplementary materials.

## References

[1] Calder P.C., Burdge G.C. (2004). Fatty Acids in Bioactive Lipids.

[2] Georgiadi A., Kersten S. (2012). Mechanisms of Gene Regulation by Fatty Acids. Adv. Nutr..

[3] Kimura I., Ichimura A., Ohue-Kitano R., Igarashi M. (2020). Free Fatty Acid Receptors in Health and Disease. Physiol. Rev..

[4] Li Z., Xu X., Huang W., Qian H. (2018). Free Fatty Acid Receptor 1 (FFAR1) as an Emerging Therapeutic Target for Type 2 Diabetes Mellitus: Recent Progress and Prevailing Challenges. Med. Res. Rev..

[5] Ulven T., Christiansen E. (2015). Dietary fatty acids and their potential for controlling metabolic diseases through activation of FFA4/GPR120. Annu. Rev. Nutr..

[6] Ghislain J., Poitout V. (2021). Targeting lipid GPCRs to treat type 2 diabetes mellitus—Progress and challenges. Nat. Rev. Endocrinol..

[7] Bolognini D., Dedeo D., Milligan G. (2021). Metabolic and inflammatory functions of short-chain fatty acid receptors. Curr. Opin. Endocr. Metab. Res..

[8] Wilding J.P. (2007). The importance of free fatty acids in the development of Type 2 diabetes. Diabet. Med..

[9] Boden G. (2011). Obesity, insulin resistance and free fatty acids. Curr. Opin. Endocrinol. Diabetes Obes..

[10] Sobczak A.I.S., Blindauer C.A., Stewart A.J. (2019). Changes in plasma free fatty acids associated with type-2 diabetes. Nutrients.

[11] Reaven G.M., Hollenbeck C., Jeng C.Y., Wu M.S., Chen Y.D. (1988). Measurement of plasma glucose, free fatty acid, lactate, and insulin for 24 h in patients with NIDDM. Diabetes.

[12] Clore J.N., Allred J., White D., Li J., Stillman J. (2002). The role of plasma fatty acid composition in endogenous glucose production in patients with type 2 diabetes mellitus. Metabolism.

[13] Yi L., He J., Liang Y., Yuan D., Gao H., Zhou H. (2007). Simultaneously quantitative measurement of comprehensive profiles of esterified and non-esterified fatty acid in plasma of type 2 diabetic patients. Chem. Phys. Lipids.

[14] Liu L., Li Y., Guan C., Li K., Wang C., Feng R., Sun C. (2010). Free fatty acid metabolic profile and biomarkers of isolated post-challenge diabetes and type 2 diabetes mellitus based on GC–MS and multivariate statistical analysis. J. Chromatogr. B.

[15] Grapov D., Adams S.H., Pedersen T.L., Garvey W.T., Newman J.W. (2012). Type 2 Diabetes Associated Changes in the Plasma Non-Esterified Fatty Acids, Oxylipins and Endocannabinoids. PLoS ONE.

[16] Lu Y., Wang Y., Ong C.-N., Subramaniam T., Choi H.W., Yuan J.-M., Koh W.-P., Pan A. (2016). Metabolic signatures and risk of type 2 diabetes in a Chinese population: An untargeted metabolomics study using both LC-MS and GC-MS. Diabetologia.

[17] Ma X.-L., Meng L., Li L.-L., Ma L.-N., Mao X.-M. (2018). Plasma Free Fatty Acids Metabolic Profile Among Uyghurs and Kazaks With or Without Type 2 Diabetes Based on GC-MS. Exp. Clin. Endocrinol. Diabetes.

[18] Kokotou M.G., Kokotos A.C., Gkikas D., Mountanea O.G., Mantzourani C., Almutairi A., Lei X., Ramanadham S., Politis P.K., Kokotos G. (2020). Saturated Hydroxy Fatty Acids Exhibit a Cell Growth Inhibitory Activity and Suppress the Cytokine-Induced β-Cell Apoptosis. J. Med. Chem..

[19] Batsika C.S., Mantzourani C., Gkikas D., Kokotou M.G., Mountanea O.G., Kokotos C.G., Politis P.K., Kokotos G. (2021). Saturated Oxo Fatty Acids (SOFAs): A Previously Unrecognized Class of Endogenous Bioactive Lipids Exhibiting a Cell Growth Inhibitory Activity. J. Med. Chem..

[20] Kokotou M.G., Mantzourani C., Bourboula A., Mountanea O.G., Kokotos G. (2020). A Liquid Chromatography-High Resolution Mass Spectrometry (LC-HRMS) Method for the Determination of Free Hydroxy Fatty Acids in Cow and Goat Milk. Molecules.

[21] Kokotou M.G., Batsika C.S., Mantzourani C., Kokotos G. (2021). Free Saturated Oxo Fatty Acids (SOFAs) and Ricinoleic Acid in Milk Determined by a Liquid Chromatography-High-Resolution Mass Spectrometry (LC-HRMS) Method. Metabolites.

[22] Zhu Q.-F., An N., Feng Y.-Q. (2020). In-Depth Annotation Strategy of Saturated Hydroxy Fatty Acids Based on Their Chromatographic Retention Behaviors and MS Fragmentation Patterns. Anal. Chem..

[23] Christinat N., Morin-Rivron D., Masoodi M. (2017). High-Throughput Quantitative Lipidomics Analysis of Nonesterified Fatty Acids in Plasma by LC-MS. Serum/Plasma Proteomics.

[24] Kokotou M.G., Mantzourani C., Kokotos G. (2020). Development of a Liquid Chromatography–High Resolution Mass Spectrometry Method for the Determination of Free Fatty Acids in Milk. Molecules.

[25] Sergeant S., Ruczinski I., Ivester P., Lee T.C., Morgan T.M., Nicklas B.J., Mathias R.A., Chilton F.H. (2016). Impact of methods used to express levels of circulating fatty acids on the degree and direction of associations with blood lipids in humans. Br. J. Nutr..

[26] Costa C.G., Dorland L., Holwerda U., De Almeida I.T., Poll-The B.T., Jakobs C., Duran M. (1998). Simultaneous analysis of plasma free fatty acids and their 3-hydroxy analogs in fatty acid beta-oxidation disorders. Clin. Chem..

[27] Chen Z., Wu Y., Shrestha R., Gao Z., Zhao Y., Miura Y., Tamakoshi A., Chiba H., Hui S.-P. (2018). Determination of total, free and esterified short-chain fatty acid in human serum by liquid chromatography-mass spectrometry. Ann. Clin. Biochem..

[28] Quehenberger O., Armando A.M., Brown A.H., Milne S.B., Myers D.S., Merrill A.H., Bandyopadhyay S., Jones K.N., Kelly S., Shaner R.L. (2010). Lipidomics reveals a remarkable diversity of lipids in human plasma. J. Lipid Res..

[29] Seo C., Yoon J., Rhee Y., Kim J.J., Nam S.J., Lee W., Lee G., Yee S.T., Paik M.J. (2015). Simultaneous analysis of seven 2-hydroxy fatty acids as tert-butyldimethylsilyl derivatives in plasma by gas chromatography-mass spectrometry. Biomed. Chromatogr..

[30] Yore M.M., Syed I., Moraes-Vieira P.M., Zhang T., Herman M.A., Homan E.A., Patel R.T., Lee J., Chen S., Peroni O.D. (2014). Discovery of a Class of Endogenous Mammalian Lipids with Anti-Diabetic and Anti-inflammatory Effects. Cell.

[31] Hama H. (2010). Fatty acid 2-Hydroxylation in mammalian sphingolipid biology. Biochim. Biophys. Acta (BBA) Mol. Cell Biol. Lipids.

[32] Haghikia A., Jörg S., Duscha A., Berg J., Manzel A., Waschbisch A., Hammer A., Lee D.-H., May C., Wilck N. (2016). Dietary Fatty Acids Directly Impact Central Nervous System Autoimmunity via the Small Intestine. Immunity.

[33] Saresella M., Marventano I., Barone M., La Rosa F., Piancone F., Mendozzi L., D’Arma A., Rossi V., Pugnetti L., Roda G. (2020). Alterations in Circulating Fatty Acid Are Associated With Gut Microbiota Dysbiosis and Inflammation in Multiple Sclerosis. Front. Immunol..

[34] Omori K., Katakami N., Yamamoto Y., Ninomiya H., Takahara M., Matsuoka T.-A., Bamba T., Fukusaki E., Shimomura I. (2019). Identification of Metabolites Associated with Onset of CAD in Diabetic Patients Using CE-MS Analysis: A Pilot Study. J. Atheroscler. Thromb..

[35] Suhre K., Meisinger C., Döring A., Altmaier E., Belcredi P., Gieger C., Chang D., Milburn M.V., Gall W.E., Weinberger K.M. (2010). Metabolic Footprint of Diabetes: A Multiplatform Metabolomics Study in an Epidemiological Setting. PLoS ONE.

[36] Menni C., Fauman E., Erte I., Perry J.R., Kastenmüller G., Shin S.Y., Petersen A.K., Hyde C., Psatha M., Ward K.J. (2013). Biomarkers for type 2 diabetes and impaired fasting glucose using a nontargeted metabolomics approach. Diabetes.

[37] Poudyal H., Brown L. (2015). Should the pharmacological actions of dietary fatty acids in cardiometabolic disorders be classified based on biological or chemical function?. Prog. Lipid Res..

