# Supplementary material for: Lipidomics Analysis of Free Fatty Acids in Human Plasma of Healthy and Diabetic Subjects by Liquid Chromatography-High Resolution Mass Spectrometry (LC-HRMS)
Source: Biomedicines. 2022 May 20;10(5):1189. doi: 10.3390/biomedicines10051189 (PMC9138513; doi:10.3390/biomedicines10051189)
Supplement: Supplementary file 1 [file biomedicines-10-01189-s001.zip › biomedicines-1713255-supplementary.pdf]

SUPPLEMENTARY MATERIALS

Lipidomics analysis of free fatty acids in human plasma of healthy and diabetic subjects by liquid chromatic-raphy-high resolution mass spectrometry (LC-HRMS)

Maroula G. Kokotou[1,2,3], Christiana Mantzourani[1,3], Charikleia S. Batsika[1,3], Olga G. Mountanea[1,3], Ioanna Eleftheriadou[3,4], Ourania Kosta[4], Nikolaos Tentolouris[3,4], George Kokotos[1,3,*]

[1]Department of Chemistry, National and Kapodistrian University of Athens, Panepistimiopolis, Athens 15771, Greece

[2]Laboratory of Chemistry, Department of Food Science and Human Nutrition, Agricultural University of Athens, Iera Odos 75, Athens 11855, Greece

[3]Center of Excellence for Drug Design and Discovery, National and Kapodistrian University of Athens, Athens 15771, Greece

[4]Diabetes Center, First Department of Propaedeutic and Internal Medicine, Medical School, National and Kapodistrian University of Athens, Laiko General Hospital, Athens 15772, Greece

Table of contents

1. Table 1S. Demographic and clinical characteristics of the participants.
2. Table 2S. List of analytes together with their exact masses $[M-H]^-$, their chromatographic retention times R_t, and their limits of detection (LOD) and quantification (LOQ).
3. Table 3S. Accuracy (recovery %) and precision data (RSD %) in spiked plasma samples for SHFAs.
4. Table 4S. Accuracy (recovery %) and precision data (RSD %) in spiked plasma samples for SOFAs.
5. Table 5S. Accuracy (recovery %) and precision data (RSD %) in spiked plasma samples for FAs.
6. Table 6S. Percentage of total FFAs analyzed.
7. Figure 1S. Multi sample analysis of medium-chain fatty acids (C6:0- C12:0) (A) T1D and (B) T2D.

8. Figure 2S. Extracted ion chromatograms (EICs) of FAs in a standard solution (500 ng/mL).
9. Figure 3S. Comparison of plasma concentrations (nmol/mL) of 3HSA, 2HSA, C13:0, C14:0, C15:0, C16:0, C16:1, C17:0, C17:1, C18:0, C18:1, C18:2, C18:3, C20:0, C20:1, C20:4, C20:5, C22:1, C22:4 and C24:1 between healthy controls and T2D and T1D patients. Graphs were created using GraphPad Prism 9.2.0. One-way ANOVA statistical analysis was performed for each separate set comparing to control. ns: $p > 0.05$. * $p < 0.05$, ** $p < 0.01$, *** $p < 0.001$, **** $p < 0.0001$.

Table S1. Demographic and clinical characteristics of the participants.

	Control samples	Type 1 DM	Type 2 DM
Female/male, n (%)	14 (50.0)/14 (50.0)	10 (71.4)/4 (28.6)	12 (41.4)/17 (58.6)
Age (years)	56.4 ± 18.9	42.9 ± 14.2	67.9 ± 12.8
Body mass index (Kg/m^2)	28.2 ± 7.6	24.2 ± 3.4	30.0 ± 5.9
Fasting glucose (mg/dL)	90.3 ± 10.4	128.1 ± 70.2	135.1 ± 49.1
HbA1c (%)	5.4 ± 0.85	7.6 ± 1.7	6.7 ± 1.1
Total cholesterol (mg/dL)	177.0 ± 47.0	181.9 ± 24.9	133.9 ± 35.4
HDL cholesterol (mg/dL)	57.4 ± 15.4	71.3 ± 17.5	42.1 ± 14.2
LDL cholesterol (mg/dL)	95.0 ± 51.4	94.1 ± 27.5	64.4± 31.5
Triglycerides (mg/dL)	112.8 ± 50.8	82.5 ± 54.4	124.8 ± 69.5
Statin treatment (yes), n (%)	0 (0)	0 (0)	11 (37.9)

DM: diabetes mellitus. Data are shown as mean standard deviation or as n (%).

Table S2. List of analytes together with their exact masses $[M\text{-}H]^-$, their chromatographic retention times R_t, and their limits of detection (LOD) and quantification (LOQ).

Analyte	$[M\text{-}H]^-$	R_t (min)	LOD (ng/mL)	LOQ (ng/mL)
3HCA [1]	187.1340	3.050	0.3	1.0
3HLA [1]	215.1653	3.894	0.5	1.4

3HMA [1]	243.1966	4.623	0.6	1.7
3HPDA [1]	257.2122	5.035	0.6	1.9
16HPA [1]	271.2279	4.301	0.9	2.6
11HPA [1]	271.2279	4.384	0.5	1.6
10HPA [1]	271.2279	4.430	0.5	1.2
9HPA [1]	271.2279	4.636	0.6	1.7
8HPA [1]	271.2279	4.487	0.5	1.2
7HPA [1]	271.2279	4.636	0.8	2.4
6HPA	271.2279	4.739	0.7	1.3
3HPA [1]	271.2279	5.337	0.5	1.4
2HPA [1]	271.2279	5.579	0.3	0.8
12HSA [1]	299.2592	5.010	0.5	1.4
11HSA	299.2592	5.060	0.6	1.3
10HSA [1]	299.2592	5.106	0.4	1.1
7HSA [1]	299.2592	5.210	0.4	1.1
8HSA [1]	299.2592	5.295	0.5	1.0
9HSA [1]	299.2592	5.321	0.6	1.8
6HSA	299.2592	5.426	0.7	2.1
3HSA [1]	299.2592	6.010	0.3	0.9
2HSA [1]	299.2592	6.229	0.1	0.4
14OPA [2]	269.2122	4.424	0.3	1.0
10OPA [2]	269.2122	4.552	0.5	1.4
9OPA [2]	269.2122	4.608	0.3	1.0
8OPA [2]	269.2122	4.780	0.5	1.4
7OPA [2]	269.2122	4.877	0.5	1.4
6OPA [2]	269.2122	4.762	0.5	1.4
5OPA	269.2122	5.042	0.6	1.8
16OSA [2]	297.2435	5.056	0.8	2.4
12OSA [2]	297.2435	5.138	0.5	1.4
10OSA [2]	297.2435	5.220	0.5	1.4
9OSA [2]	297.2435	5.348	0.5	1.4
8OSA [2]	297.2435	5.276	0.5	1.4
7OSA [2]	297.2435	5.439	0.8	2.4
6OSA [2]	297.2435	5.548	0.8	2.4
5OSA	297.2435	5.702	0.9	1.8
4OSA	297.2435	5.889	0.8	1.9
3OSA	297.2435	6.221	0.9	2.1
Caproic acid (C6:0) [3]	115.0765	2.101	0.5	1.5
Heptanoic acid (C7:0)	129.0921	2.704	0.5	1.3
Caprylic acid (C8:0) [3]	143.1078	3.235	0.5	1.1
Nonanoic acid (C9:0)	157.1234	3.691	0.5	1.2
Capric acid (C10:0) [3]	171.1391	4.123	0.5	1.7

Undecanoic acid (C11:0)	185.1547	4.519	0.5	1.6
Lauric acid (C12:0) [3]	199.1704	4.908	0.6	1.8
Tridecanoic acid (C13:0)	213.1860	5.270	0.7	2.2
Myristic acid (C14:0) [3]	227.2017	5.632	0.6	1.8
Myristoleic acid (C14:1) [3]	225.1850	5.120	0.6	1.8
Pentadecanoic acid (C15:0) [3]	241.2173	5.957	0.8	2.4
Palmitic acid (C16:0) [3]	255.2330	6.290	0.9	2.3
cis-9-Palmitoleic acid (C16:1) [3]	253.2173	5.767	1.6	4.8
Margaric acid (C17:0) [3]	269.2486	6.557	0.8	2.4
cis-10-Heptadecenoic acid (C17:1) [3]	267.2330	6.201	0.8	2.4
Stearic acid (C18:0) [3]	283.2643	6.825	0.9	2.8
Oleic acid (C18:1) [3]	281.2486	6.368	0.7	2.3
Linoleic acid (C18:2) [3]	279.2330	6.010	0.6	1.8
Linolenic acid (C18:3) [3]	277.2173	5.710	0.6	1.8
Nonadecanoic acid (C19:0)	297.2799	7.067	0.7	1.9
Arachidic acid (C20:0) [3]	311.2956	6.975	0.8	2.4
cis-11-Eicosenoic acid (C20:1)	309.2799	6.872	0.8	2.4
Bishomo-γ-linolenic acid (C20:3) [3]	305.2486	6.140	0.6	1.8
Arachidonic acid (C20:4) [3]	303.2330	5.889	0.6	1.8
cis-5,8,11,14,17-Eicosapentaenoic acid (C20:5) [3]	301.2173	5.613	0.6	1.8
Phytanic acid (3,7,11,15-tetramethyl hexadecenoic acid)	311.2956	6.975	0.8	2.4
Heneicosanoic acid (C21:0)	325.3112	7.498	0.8	2.4
Behenic acid (C22:0)	339.3269	7.683	0.8	2.4
cis-13-Docosenoic acid (C22:1)	337.3112	7.301	0.8	2.4
Adrenic acid (C22:4)	331.2643	6.329	0.8	2.4
cis-7,10,13,16,19-Docosapentaenoic acid (C22:5) [3]	329.2486	6.131	0.4	1.2
cis-4,7,10,13,16,19-Docosahexaenoic acid (C22:6) [3]	327.2330	5.921	0.4	1.2
Tricosanoic acid (C23:0)	353.3425	7.846	0.7	1.4
Nervonic acid (C24:1)	365.3425	7.680	0.8	2.2

Ricinoleic acid [2]	297.2435	4.754	0.6	2.0

Table 3S. Accuracy (recovery %) and precision data (RSD %) in spiked plasma samples for SHFAs.

Analyte	Spike level 10 ng/mL		Spike level 100 ng/mL		Spike level 500 ng/mL	
	Recovery (%R)	RSD (%)	Recovery (%R)	RSD (%)	Recovery (%R)	RSD (%)
3HCA	85	9.36	86	8.97	94	3.80
3HLA	90	9.24	83	3.71	94	1.49
3HMA	81	1.11	79	2.65	91	3.90
3HPDA	81	11.60	78	6.01	98	2.46
16HPA	93	0.25	87	8.00	104	11.42
11HPA	72	3.72	79	2.67	91	3.75
10HPA	92	13.92	85	9.25	96	4.53
9HPA	74	5.40	78	0.53	98	9.96
8HPA	70	3.25	81	14.24	94	6.68
7HPA	83	12.47	83	2.80	102	1.91
6HPA	90	10.46	85	1.89	99	6.88
3HPA	95	3.41	92	3.28	106	9.17
2HPA	93	2.85	87	6.02	91	6.34
12HSA	107	9.81	94	3.84	94	0.07
11HSA	98	1.44	85	11.56	88	5.85
10HSA	92	15.57	81	9.34	86	2.32
9HSA	76	1.12	82	1.63	87	4.94
8HSA	85	6.02	78	7.75	88	10.11
7HSA	84	9.89	86	2.82	83	0.04
6HSA	78	1.40	81	2.49	91	18.07
3HSA	98	10.44	89	8.84	91	7.17
2HSA	92	8.81	85	8.15	94	3.80

Table 4S. Accuracy (recovery %) and precision data (RSD %) in spiked plasma samples for SOFAs.

Analyte	Spike level 10 ng/mL		Spike level 100 ng/mL		Spike level 500 ng/mL	
	Recovery	RSD (%)	Recovery	RSD (%)	Recovery	RSD (%)

	(%R)		(%R)		(%R)	
14OPA	87	9.84	84	7.32	81	6.45
10OPA	101	6.64	81	3.21	86	7.67
9OPA	102	3.66	81	4.12	90	0.94
8OPA	101	3.66	83	2.87	86	3.50
7OPA	90	2.32	80	3.12	83	9.07
6OPA	77	7.02	85	8.51	81	9.30
5OPA	102	9.99	88	10.01	86	15.49
16OSA	71	0.82	88	1.34	74	4.86
12OSA	70	9.78	80	5.18	80	0.86
10OSA	79	8.83	82	9.52	88	13.59
9OSA	91	2.33	86	3.01	83	3.59
8OSA	70	12.41	81	4.57	88	5.27
7OSA	80	10.15	80	10.24	83	10.12
6OSA	88	10.09	84	6.75	87	5.30
5OSA	76	2.76	84	5.81	84	7.85
4OSA	93	6.14	81	7.23	85	13.60
3OSA	70	0.92	85	8.12	77	6.27

Table 5S. Accuracy (recovery %) and precision data (RSD %) in spiked plasma samples for FAs.

Analyte	Spike level 10 ng/mL		Spike level 100 ng/mL		Spike level 500 ng/mL	
	Recovery (%R)	RSD (%)	Recovery (%R)	RSD (%)	Recovery (%R)	RSD (%)
Caproic acid (C6:0)	101	12.39	88	10.68	101	5.92
Heptanoic acid (C7:0)	96	0.74	88	0.53	86	10.52
Caprylic acid (C8:0)	92	6.27	79	7.73	97	6.17
Nonanoic acid (C9:0)	100	4.90	95	5.87	90	5.62
Capric acid (C10:0)	90	15.05	86	16.63	91	4.00
Undecanoic acid (C11:0)	80	5.74	84	5.34	100	8.88
Lauric acid (C12:0)	97	11.37	99	0.88	95	8.21
Tridecanoic acid	92	0.59	83	2.28	85	3.84

(C13:0)						
Myristic acid (C14:0)	97	1.82	99	0.85	89	12.84
Myristoleic acid (C14:1)	98	5.08	97	9.47	91	7.28
Pentadecanoic acid (C15:0)	87	0.07	95	6.01	91	9.94
Palmitic acid (C16:0)	97	1.74	93	2.33	90	8.50
cis-9-Palmitoleic acid (C16:1)	93	0.78	94	0.22	97	6.85
Margaric acid (C17:0)	98	6.83	99	5.27	89	4.10
cis-10-Heptadecenoic acid (C17:1)	92	0.24	100	0.61	101	0.77
Stearic acid (C18:0)	91	0.84	95	6.33	100	0.45
Oleic acid (C18:1)	95	8.34	94	10.36	83	2.51
Linoleic acid (C18:2)	96	4.73	94	1.62	87	2.13
Linolenic acid (C18:3)	96	2.61	97	0.83	82	4.63
Nonadecanoic acid (C19:0)	91	9.60	90	2.48	84	3.00
Arachidic acid (C20:0)	103	0.74	88	2.61	102	7.03
cis-11-Eicosenoic acid (C20:1)	101	1.85	86	3.27	89	11.74
Bishomo-γ-linolenic acid (C20:3)	93	1.08	99	3.39	92	4.37
Arachidonic acid (C20:4)	93	1.97	95	4.60	105	4.21
cis-5,8,11,14,17-Eicosapentaenoic acid (C20:5)	94	1.42	82	8.36	80	5.26
Phytanic acid (3,7,11,15-tetramethyl hexadecenoic	87	7.09	94	7.67	91	0.04

acid)						
Heneicosanoic acid (C21:0)	84	3.56	84	4.02	88	9.15
Behenic acid (C22:0)	97	1.44	86	4.62	88	2.71
cis-13-Docosenoic acid (C22:1)	100	2.03	95	3.80	84	6.00
Adrenic acid (C22:4)	93	2.50	88	3.27	89	1.87
cis-7,10,13,16,19-Docosapentaenoic acid (C22:5)	100	7.52	96	2.70	75	16.30
cis-4,7,10,13,16,19-Docosahexaenoic acid (C22:6)	90	2.07	100	8.20	105	1.46
Tricosanoic acid (C23:0)	79	4.04	95	4.19	84	1.34
Nervonic acid (C24:1)	94	3.63	103	9.18	108	0.59
Ricinoleic acid	91	10.65	96	7.50	92	0.24

Table 6S. Percentage of total FFAs analyzed.

FA	Control plasma sample (%)	T2D plasma sample (%)	T1D plasma sample (%)
C6:0	0.55	0.72	0.78
C7:0	0.03	0.03	0.04
C8:0	0.12	0.16	0.16
C9:0	0.22	0.29	0.43
C10:0	0.07	0.12	0.09
C11:0	-	-	-
C12:0	0.17	1.75	0.15
C13:0	0.01	0.02	0.01
C14:0	3.51	4.05	3.81
C14:1	0.16	0.17	0.19
C15:0	0.09	0.13	0.20
C16:0	20.72	24.12	22.18
C16:1	3.12	2.76	3.03
C17:0	0.97	1.15	0.85
C17:1	0.26	0.29	0.25

C18:0	6.54	5.99	7.09
C18:1	13.36	14.94	14.32
C18:2	46.69	39.68	43.88
C18:3	0.39	0.49	0.46
Ricinoleic acid	0.01	0.02	0.01
C19:0	0.01	0.01	0.01
C20:0	0.08	0.09	0.07
C20:1	0.30	0.37	0.24
C20:3	-	-	-
C20:4	0.44	0.48	0.37
C20:5	0.25	0.39	0.25
Phytanic acid	0.01	0.02	0.02
C21:0	-	-	-
C22:0	-	-	-
C22:1	0.02	0.02	0.01
C22:4	0.87	0.93	0.52
C22:5	0.72	0.39	0.33
C22:6	-	-	-
C23:0	-	-	-
C24:1	0.18	0.18	0.12
SHFAs	0.13	0.22	0.12
SOFAs	0.01	0.03	0.02

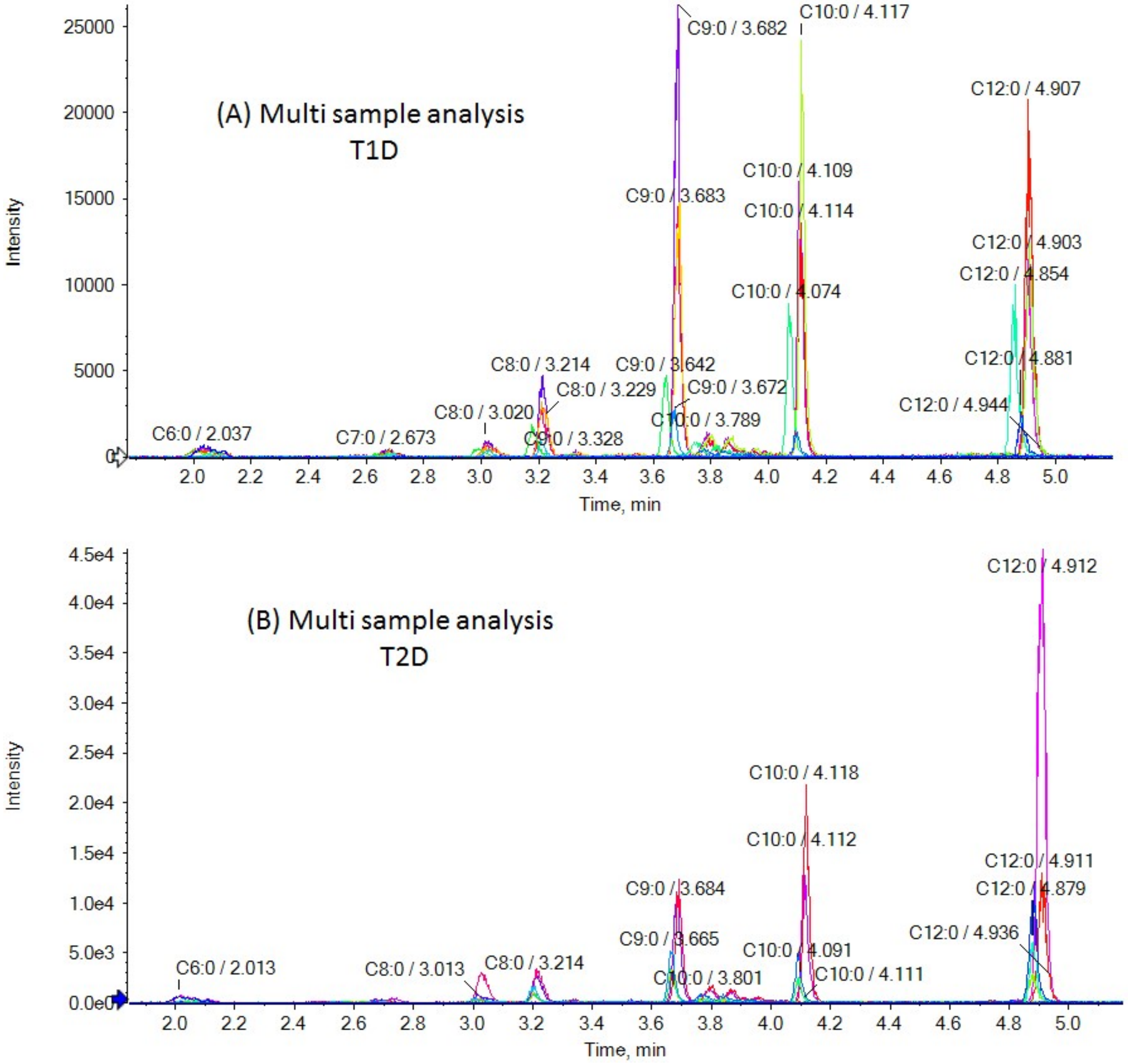

Figure S1. Multi sample analysis of medium-chain fatty acids (C6:0- C12:0) (**A**) T1D and (**B**) T2D.

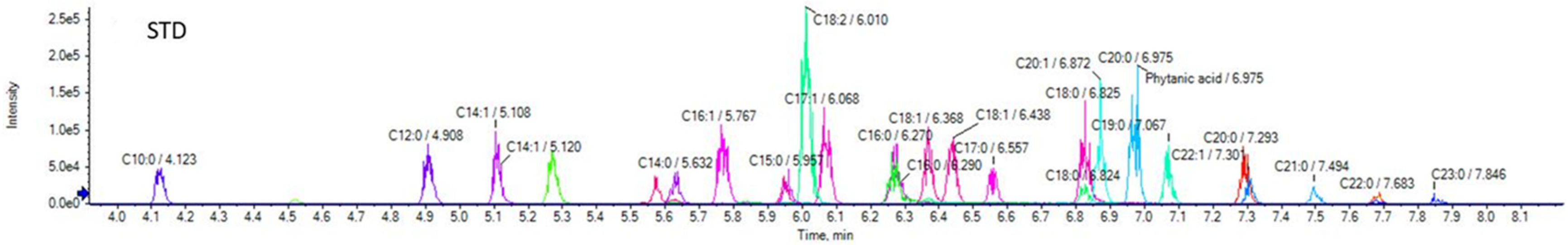

Figure S2. Extracted ion chromatograms (EICs) of FAs in a standard solution (500 ng/mL).

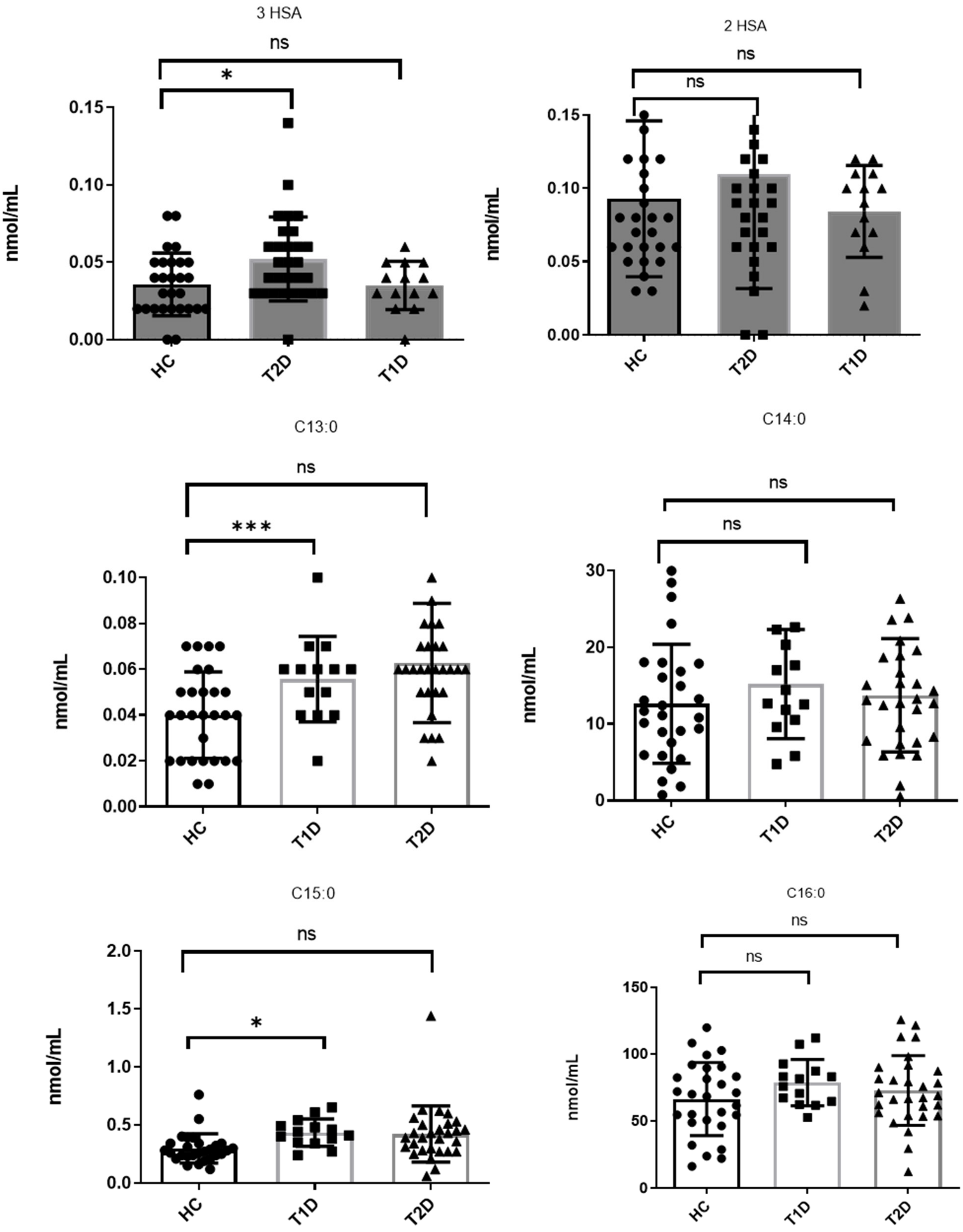
3 HSA
2 HSA
C13:0
C14:0
C15:0
C16:0
nmol/mL
HC
T2D
T1D
ns
*
***

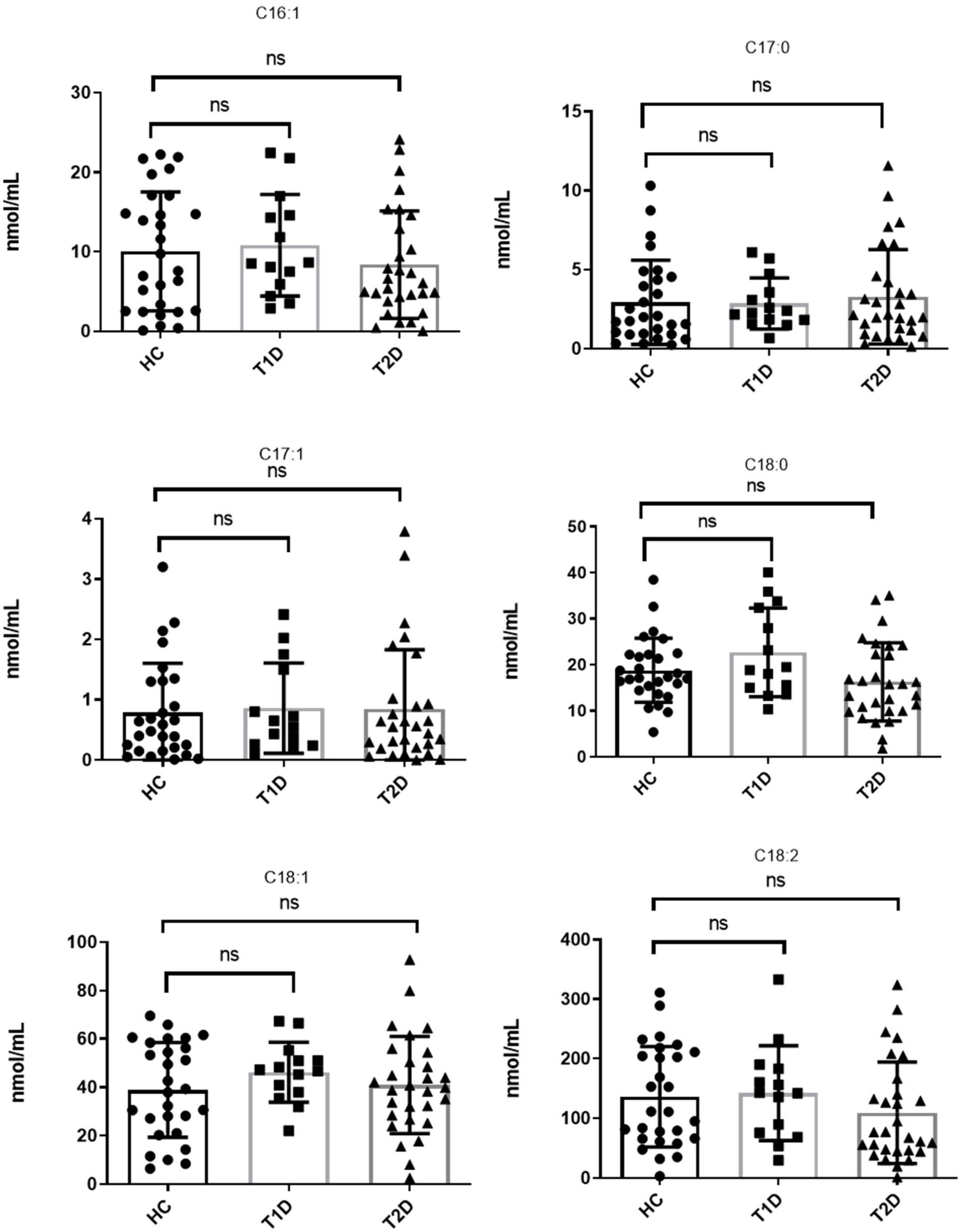
C16:1
ns
ns
30
20
10
0
nmol/mL
HC
T1D
T2D
C17:0
ns
ns
15
10
5
0
nmol/mL
HC
T1D
T2D
C17:1
ns
ns
4
3
2
1
0
nmol/mL
HC
T1D
T2D
C18:0
ns
ns
50
40
30
20
10
0
nmol/mL
HC
T1D
T2D
C18:1
ns
ns
100
80
60
40
20
0
nmol/mL
HC
T1D
T2D
C18:2
ns
ns
400
300
200
100
0
nmol/mL
HC
T1D
T2D

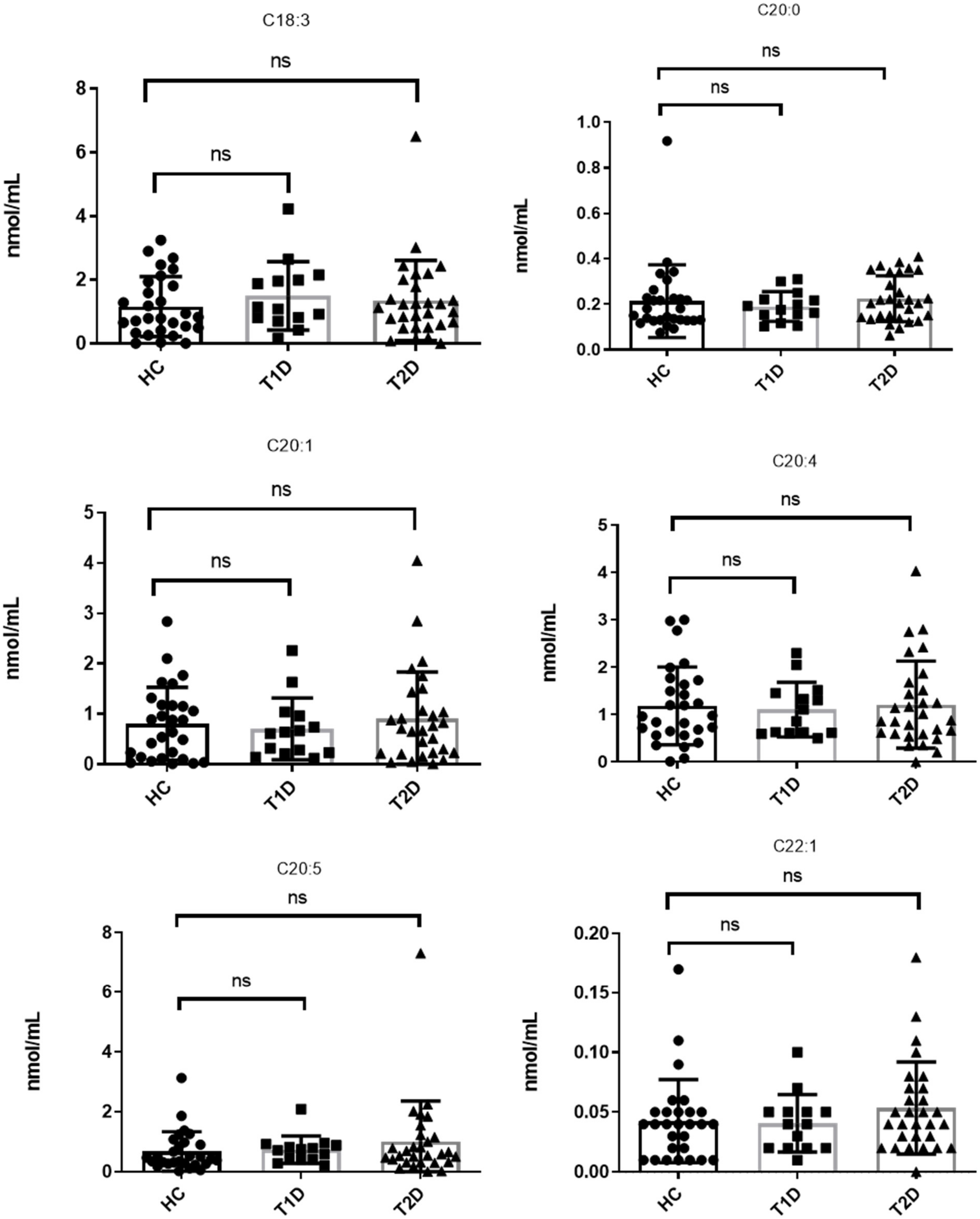
C18:3
C20:0
C20:1
C20:4
C20:5
C22:1
ns
nmol/mL
HC
T1D
T2D

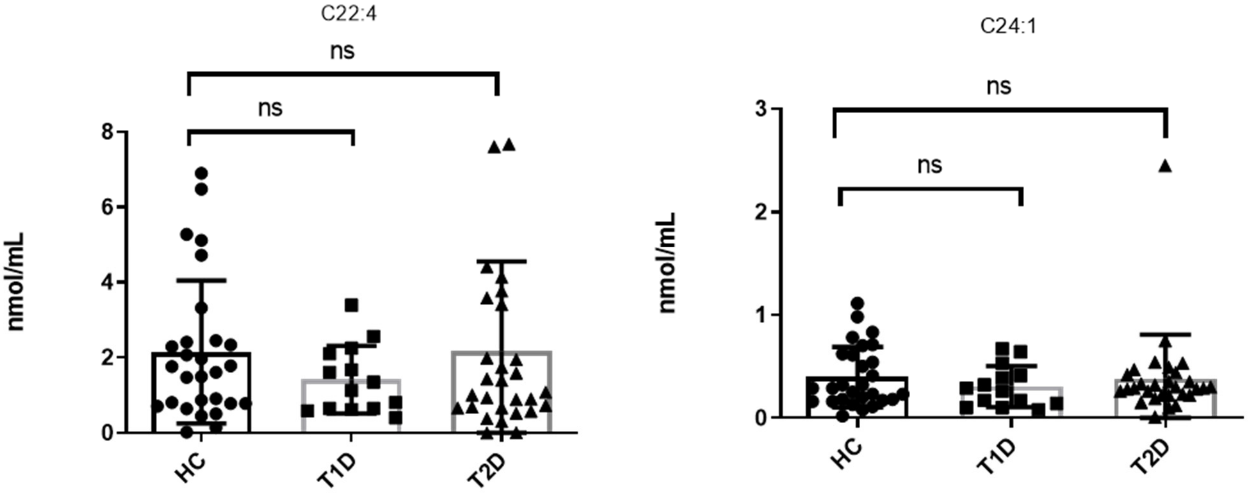

Figure S3. Comparison of plasma concentrations (nmol/mL) of 3HSA, 2HSA, C13:0, C14:0, C15:0, C16:0, C16:1, C17:0, C17:1, C18:0, C18:1, C18:2, C18:3, C20:0, C20:1, C20:4, C20:5, C22:1, C22:4 and C24:1 between healthy controls and T2D and T1D patients. Graphs were created using GraphPad Prism 9.2.0. One-way ANOVA statistical analysis was performed for each separate set comparing to control. ns: $p > 0.05$. * $p < 0.05$, ** $p < 0.01$, *** $p < 0.001$, **** $p < 0.0001$.

References

1. Kokotou, M.G.; Mantzourani, C.; Bourboula, A.; Mountanea, O.G.; Kokotos, G. A Liquid Chromatography-High Resolution Mass Spectrometry (LC-HRMS) Method for the Determination of Free Hydroxy Fatty Acids in Cow and Goat Milk. *Molecules* **2020**, *25*, 3947. https://doi.org/10.3390/molecules25173947.
2. Kokotou, M.G.; Batsika, C.S.; Mantzourani, C.; Kokotos, G. Free Saturated Oxo Fatty Acids (SOFAs) and Ricinoleic Acid in Milk Determined by a Liquid Chromatography-High-Resolution Mass Spectrometry (LC-HRMS) Method. *Metabolites* **2021**, *11*, 46. https://doi.org/10.3390/metabo11010046.
3. Kokotou, M.G.; Mantzourani, C.; Kokotos, G. Development of a Liquid Chromatography–High Resolution Mass Spectrometry Method for the Determination of Free Fatty Acids in Milk. *Molecules* **2020**, *25*, 1548. https://doi.org/10.3390/molecules25071548.
